# Children’s value-based decision making

**DOI:** 10.1038/s41598-022-09894-3

**Published:** 2022-04-08

**Authors:** Karen E. Smith, Seth D. Pollak

**Affiliations:** Department of Psychology and Waisman Center, University of Wisconsin – Madison, 1500 Highland Ave, Rm 392, Madison, WI 53705 USA

**Keywords:** Emotion, Learning and memory, Reward, Psychology, Human behaviour

## Abstract

To effectively navigate their environments, infants and children learn how to recognize events predict salient outcomes, such as rewards or punishments. Relatively little is known about how children acquire this ability to attach value to the stimuli they encounter. Studies often examine children’s ability to learn about rewards and threats using either classical conditioning or behavioral choice paradigms. Here, we assess both approaches and find that they yield different outcomes in terms of which individuals had efficiently learned the value of information presented to them. The findings offer new insights into understanding how to assess different facets of value learning in children.

## Introduction

Learning the predictive value associated with environmental stimuli is essential to adaptive decision making^1,2^. It facilitates a wide range of behaviors across development including obtaining food, avoiding life-threatening injury, adequate seeking of protection, and effective navigation of the social world^3,4^. For these reasons, there has been a growth in research aimed at elucidating the mechanisms through which value learning emerges^5,6^. In these studies, acquisition of value information is typically assessed by pairing neutral stimuli with a salient outcome. Learning is then inferred in one of two ways: through subsequent physiological or behavioral reactivity to the neutral stimuli, or the extent to which participants use the stimuli to guide their behavioral choices^7–9^. However, it is not clear that these two approaches index similar measures of learning. To test the comparability of these approaches, we presented children with an opportunity to learn the value of stimuli and then assessed both changes to their reactivity to the new information as well as the degree to which that new information changed their decision-making.

Humans can recognize and use probabilistic relationships in their environments as early as infancy^10,11^. Indeed, probabilistic learning during early childhood has been implicated in a number of different processes, including language learning, category formation, and social behavior^12–14^. Additionally, probabilistic learning of reward and threat has been implicated in behavioral outcomes, such as the emergence of risk taking and aggression^15,16^ and a range of psychopathologies^17–19^. Together this suggests that value learning is present early in development and plays a critical role in shaping how children interact and engage with their environment.

Because early value learning has been associated with the emergence of behavioral and mental health problems, there has been interest in examining how early environments might influence the development of value learning processes. However, this literature remains inconclusive. Some evidence suggests that stress exposure and predictability in childhood are linked to the development of reward and threat learning processes^7,9,20–22,40^. Other reports indicate limited support for any association^7,23,24^. One potential explanation for these discrepancies is these studies use a variety of different paradigms to assess children’s learning, which may tap very different components of these processes.

Specifically, the different paradigms used in studies of children’s value learning may not represent comparable measures of learning. The types of paradigms employed to assess children’s value learning are typically either classical Pavlovian or instrumental conditioning paradigms. In both of these paradigms, neutral cues (or unsigned value signals) are paired with appetitive or aversive reinforcers. In classical Pavlovian conditioning, learning is inferred based on whether or not an organism demonstrates physiological and behavioral responses to the neutral cue (i.e., heart rate and skin conductance responses, freezing, reaction times). In instrumental paradigms, learning is inferred based on whether an organism executes the expected behavioral response to the neutral cue. For example, if an organism presses a lever to avoid administration of an aversive shock, it is assumed they have learned predictive value of the neutral stimulus^1,16,25^. Although these two paradigms are both used to assess value learning in children, there are potentially important differences between them. Classical conditioning tasks use physiological and behavioral responses elicited directly by the neutral cue to assess whether the child has linked some value to a stimulus, but do not determine whether that information is directly or meaningfully translated into guiding decision-making or behavior^25,26^. Instrumental tasks provide insight into goal directed behaviors. However, they are dependent on inferences of learning based upon whether a child executes a behavioral response to either approach an appetitive reinforcer or avoid an aversive reinforcer^27,28^. In sum, there may be many reasons that a child decides whether or not to execute a behavioral response that are not necessarily related to whether they have acquired relevant, new information.

As one example, motivation may influence how a child behaves during assessment of learning. This potential confound between learning and motivation is illustrated by studies in which monetary (or point) rewards are used as appetitive reinforcers^7,29,30^. These approaches are predicated upon the idea that children who have learned neutral cue–reinforcer relationships will respond to the neutral cues in ways that maximize their receipt of monetary rewards. Children’s learning is then modeled based on their behavioral choices^25,31^. While it could be the case that children do not execute the expected behavioral responses because they have not learned, they also may not execute the expected responses because they are not sufficiently motivated by the monetary reward to use the information they have learned. Conversely, a loss of monetary reward may not be sufficiently salient to change behavior^32^. Comparing children’s performance across different assessments of learning can aid in illuminating whether behavior is being driven by learning or other motivational drives.

To test whether different measures of value learning identify similar groups of children as having learned, we compared children’s performance on a classical conditioned learning task and on a behavioral choice task. Children underwent a Pavlovian conditioning paradigm involving both appetitive and aversive reinforcers. Next, we assessed the extent to which they were able to use the conditioned stimuli to make decisions about whether to approach or avoid the various reinforcers. If both approaches are measuring comparable aspects of learning, children who demonstrate good associative learning on the conditioning paradigm should also be able to use that learned information to guide their approach and avoidance decisions. We assessed learning through both overt behavior and autonomic nervous system reactivity, and tested learning using a variety of different reinforcers to guard against stimulus-specific effects.

## Results

### Value learning as assessed by conditioning

**Figure 1:**
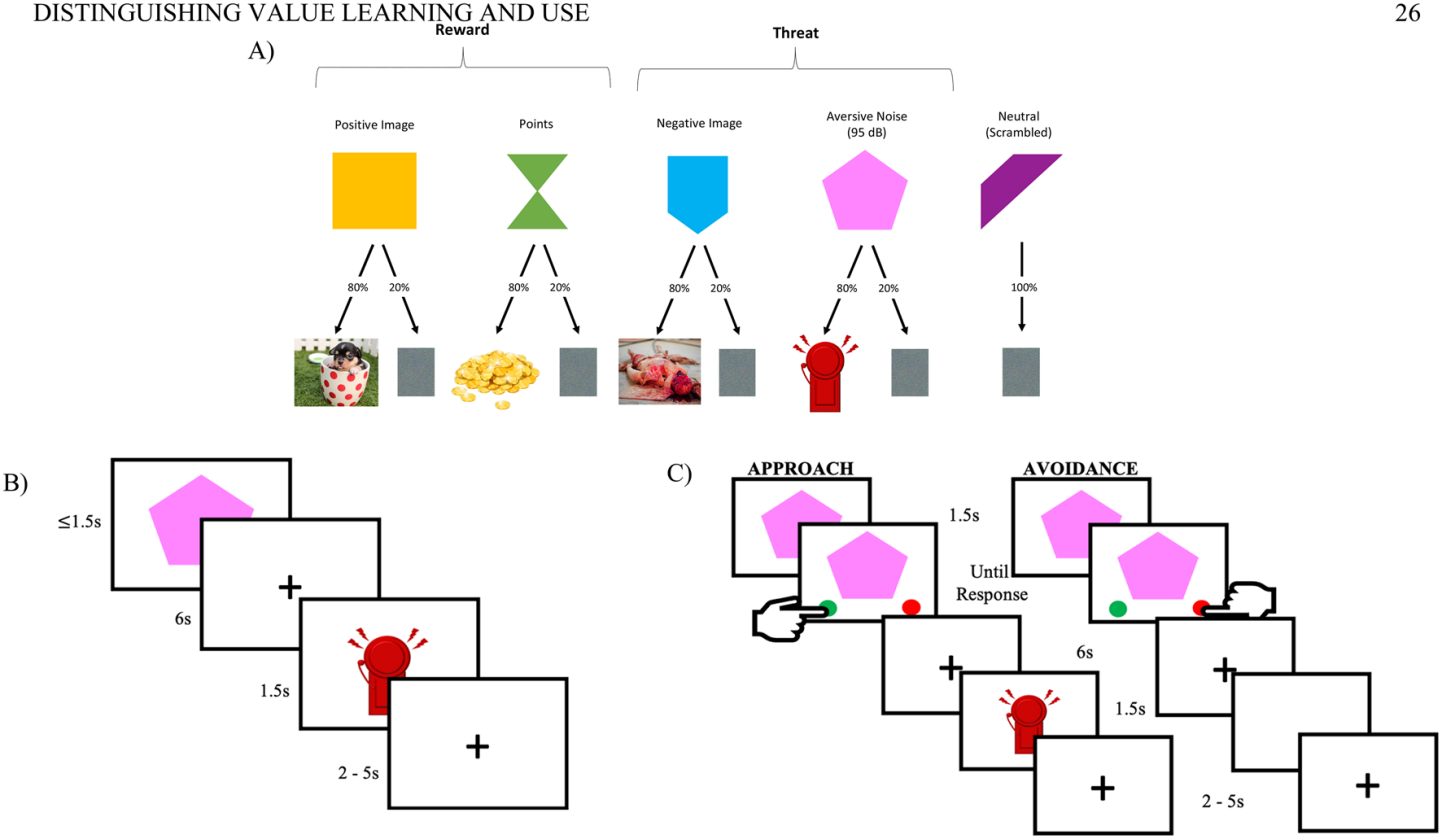
Task schematics. (**A**) Example of neutral shape—reinforcer pairings and probability ratios. Neutral shapes were paired with either a positive image, points reward, negative image, or aversive noise 80% of the time and neutral scrambled image 20% of the time. One shape was always paired with the neutral scrambled image. Images were taken from the Open Affective Standardized Image Set (OASIS; Positive Image: I256; Negative Image: I287), an open access set of images with standardized ratings of valence and arousal^50^. (**B**) Example of a trial in the conditioning task. (**C**) Example of a trial in the behavioral choice task. Pressing the green button resulted presentation of the reinforcer; pressing the red button resulted in presentation of a blank screen. Thus, pressing the green button represents an approach response and pressing the red represents an avoidance response. Figure adapted from Smith and Pollak^47^.

We began by confirming that children learned the value of the previously neutral stimulus items during conditioning (for task design see Figs. 1 and Figure S1). Learning was assessed in three ways. First, we ran an HLM model including pre-conditioning and post-conditioning rating and reinforcer type as fixed effects with random effects for reinforcer type nested within subject for Visual Analogue Scale ratings. Children rated cues paired with appetitive reinforcers more positively after conditioning and those paired with aversive reinforcers more negatively (χ^2^(4) = 15.28, p = 0.004). This was especially true for the points and aversive noise reinforcers. Second, we examined children’s modeled learning rates. Across participants, the best fit yielded a learning rate of 0.2 which is similar to those utilized in other studies^33–35^. As a tertiary measure of learning, we also examined heart rate reactivity (using IBIs) to different reinforcer trials during the conditioning task. Children demonstrated differential heart rate reactivity to the different reinforcers (χ^2^(4) = 50.27, p < 0.001) further indicative of learning during the task (for full discussion of heart rate analyses see Supplemental Materials). Including age, gender, and WASI-II score in the models did not change any of the reported effects. Further details of the analyses are reported in the Supplemental Materials Tables S1 and S2.

### Value learning as assessed by behavioral choice

We next examined children’s behavior on the behavioral choice task (Fig. 1) to determine how they used the information they learned in the conditioning task. To do so, we ran logistic HLM models with a random effect for reinforcer type nested within participant. Reinforcer type (points, positive image, aversive noise, negative image) was included as a fixed factor and whether or not the child approached was the outcome variable. As expected, children approached the appetitive reinforcers and avoided the aversive reinforcers (χ^2^(4) = 143.37, p < 0.001). As with the Visual Analogue Scale ratings, this was most pronounced for the points and aversive noise. Including age, gender, and WASI-II score did not change any of the reported effects. Further details of the analyses are reported in the Supplemental Materials.

### Comparing performance on the conditioning and behavioral choice tasks

**Figure 2:**
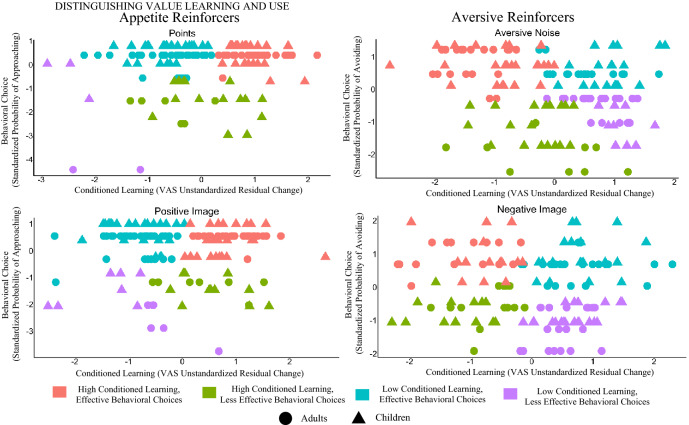
Clusters based on participants’ performance on the conditioning and behavioral choice tasks (for first five trials) for each reinforcer condition. Four comparable clusters of learning on the conditioning task and use on the behavioral choice task were identified for all reinforcer conditions suggestive of a separation between conditioned learning and behavioral choice based assessments of value information acquisition. They followed this general pattern: (1) Individuals who demonstrated high learning on the conditioning task and high use on the behavioral choice task (in red); (2) Individuals who demonstrated high learning on the conditioning task and low use on the behavioral choice task (in green); (3) Individuals who demonstrated low learning on the conditioning task and high use on the behavioral choice task (in blue); and (4) Individuals who demonstrated low learning on the conditioning task and low use on the behavioral choice task (in purple). All axes are standardized. *VAS* Visual Analogue Scale.

To determine if performance on the conditioning task and behavioral choice task identified similar groups of children as having acquired the value of the stimuli, we examined clusters of behavior using change in pre- and post-conditioning ratings of the neutral shapes and use behaviors on the approach avoidance task. Our measure of learning during the conditioning task was change in visual analogue scale ratings of the shapes measured using unstandardized residualized change scores. For the points and the positive image, more positive change is indicative of increased learning, and for the aversive noise and negative image, more negative change is indicative of greater learning. Our measure of use behaviors was children’s likelihood of demonstrating the expected behavior. Effective use of information reflects a participant choosing to approach appetitive reinforcers and avoid aversive reinforcers. Because there is an opportunity for additional learning in the behavioral choice task, analyses were run using only behavior on the first five trials of the task. If performance on the two tasks is comparable, the cluster analysis should identify two latent subgroups of behavior: one in which children demonstrate learning on the conditioning task and effective use of information on the behavioral choice task and a second in which individuals demonstrate little evidence of learning on the conditioning task and poor use of information on the behavioral choice task. Consistent with this, we identified a group of children that appeared to demonstrate higher conditioned learning and higher effective use of information as well as a group of children with lower conditioned learning and lower effective use of information. However, two additional groups of behavior were also identified: a group of children demonstrating higher conditioned learning but lower effective use of information and a group of children demonstrating lower conditioned learning and higher effective use of information. The presence of these two additional groups suggests that the two tasks do not provide comparable inferences of learning for all individuals. The four clusters were similar across the different reinforcer conditions (points, positive image, aversive noise, negative image) (Fig. 2). We also ran all cluster analyses using an alternative measure of learning (behavior derived from our computational reinforcement model) and behavior across all trials and found comparable patterns which are reported in Figures S2–S6.

### Examination of alternative hypotheses

It was possible that children did not use information they learned in the conditioning task because they had forgotten the cue reinforcer relationships by the time they completed the behavioral choice task. To test this alternative hypothesis, we assessed explicit recall for these relationships at the completion of the behavioral choice task (Figure S1). We found no evidence that an inability to recall the cue reinforcer relationships accounted for performance differences between high and low use groups for high learners (ps > 0.10; Table S3). Additionally, there was no consistent evidence that the relationship between learning and use of value information was associated with children’s age, gender, or general cognitive ability (ps > 0.10; Table S3). It was also possible that the observed pattern of behaviors across the two tasks was due to children’s relative cognitive immaturity. To assess whether our findings replicate in an adult sample, we conducted a follow-up study with young adults (n = 74) using the same experimental paradigm (see also^36^). We found comparable effects (Fig. 2), suggesting our findings are not specific to early childhood. Full methods and results for the adult follow-up study are reported in the Supplemental Materials (Table S4).

## Discussion

We tested whether classical conditioning and behavioral choice tasks identify similar groups of children as having learned a set of value information and found that they do not. Some children demonstrated learning on the conditioning task that was similarly reflected in their actions in the behavioral choice task. Yet others demonstrated evidence of learning based only on the conditioning task that was not reflected in their behavioral choices, or, conversely, showed little evidence of learning on the conditioning task but clearly used the learned value information in making their behavioral choices. Last, some children demonstrated little evidence of learning based on either tasks. This finding helps account for some reported inconsistencies in the reward and threat learning literature.

The robustness of this dissociation is supported by several lines of convergent evidence. First, we found similar evidence for this phenomenon across four distinct types of reinforcers, making it unlikely that there are stimulus-specific effects across individuals. Second, we found similar patterns using different ways to assess learning on the conditioning task, making it unlikely that the effects are specific to one type of measurement. Third, children’s using previously learned information less effectively was not accounted for by the participant’s forgetting the value information or general cognitive factors. Our findings suggest that studies assessing value (or reward/threat) learning in children should carefully consider whether the method of assessment being used aligns with the primary question of interest—neither approach is more or less accurate, but each approach assesses a distinct aspect of learning. For this reason, there is utility in using multiple tasks when examining learning and motivational processes.

Utilizing multiple assessments of learning may be especially critical when assessing individual differences in value learning processes, or the effects of experiences on the acquisition of value information. Altered value learning has been implicated in a range of psychopathologies^17–19^, and there is growing evidence altered value learning may be one mechanism through which adverse experiences early in childhood increase risk for later psychopathology and behavioral problems^9,37,38^. To date, research in this area cannot make clear claims about whether observed differences are linked to learning or differences in other motivational drives^39,40^. Some recent evidence suggests it is likely the latter^41^. Using multiple assessments can help clarify what motivational components underlie these observed behavioral differences.

Taken together, the present data indicate a need for further research examining the mechanisms underlying what transforms learned information into action in early childhood—or what prevents acquired information from being transformed into action. Of particular interest are central prefrontal-dopaminergic striatal circuits. These circuits appear to play an important role in encoding value and informing goal directed approach avoidance behaviors in instrumental learning tasks^27,42^. Dopaminergic activity has been linked to effective approach and avoidance of appetitive and aversive stimuli respectively^1,43^. Additionally, dopamine may be particularly relevant to the motivational salience of stimuli^44^. Research examining reactivity in central circuits can test whether altered reactivity in dopaminergic circuits contributes to the differential behavioral patterns observed.

Future research can refine the relationship between children’s learning and use of value information. One potentially surprising finding is that we identified children who demonstrated lower learning on the conditioning task but still executed the expected behavioral choices on the approach avoidance task. We suspect that the approach avoidance task provided some additional opportunity for learning. This is likely because children continue to be exposed to and receive feedback about the cue-reinforcer relationships across trials. Since our measures of learning are continuous and not dichotomous (learning/no learning)^16,25^, this re-exposure to the stimuli may have reactivated these representations for some participants. An alternative explanation is this is due to the fact that the instrumental nature of the approach avoidance task provides participants with increased control over the outcomes. Provision of control has been demonstrated to increase motivation during value-based tasks and increase the subjective value of reinforcers^45,46^. Thus, this increased control may have facilitated faster encoding of value information for some individuals who previously demonstrated poorer learning. The lack of substantial differences between clusters examined using behavior during the first five trials and across the entire task, could be interpreted as evidence against additional learning. However, both reactivation of prior representations of shape reinforcer relationships and facilitation of learning by increased control could have occurred rapidly, within the first five trials. Further research can further examine what may be driving this effect.

It could be argued that the differences we observe in performance across the tasks are reflective of other within-individual differences not captured by our measures of learning or memory for cue reinforcer relationships. However, we did utilize multiple measures of learning and found a similar pattern of dissociation in performance on the tasks across these convergent measures. It could also be argued that the differences we observe are a result of the two tasks indexing different forms of learning^27,28^; yet the observation that some children only approached positive stimuli and only avoided negative stimuli suggest this is not driving differential performance on the two tasks. These behavioral decisions would not have been possible if children were not applying previously learned information from the conditioning task. Finally, the current sample was primarily White and the samples for some of the low use groups are relatively small. Future research should replicate this effect in larger, more diverse samples with more representation in both high use and low use groups to reduce limitations associated with generalizability.

Overall, these data cast a new light on how to assess the processes through which children acquire critical information from stimuli in the environment and transform that information to guide their actions, broadly referred to as value-based decision making. Research aimed at further examining the factors the prevent learned information from being used in decision making can aid in our understanding of the mechanisms underlying these effects and holds potential for informing intervention and treatment for behavioral problems associated with disrupted value learning and use.

## Method

### Participants

We aimed to recruit 70 child participants (see also^47^). Final recruitment was 72 children (29 female 8 – 9 years old (M = 8.43; SD = 0.50; Race: 65.3% White Non-Hispanic; 2.8% Asian; 9.7% Black/African American; 9.7% White Hispanic; 4.2% Hispanic; 4.2% Multi-Racial; 4.2% Other). We recruited children in this age range because it appears to be the earliest period when children reliably exhibit both appetitive and aversive conditioned learning^7,48^. Children provided verbal assent, and their parents provided written informed consent. Child participants received a toy prize and their parents received $25. This study was approved by the University of Wisconsin-Madison Institutional Review Board and performed in accordance with relevant guidelines and regulations.

### Procedure

Methods are the same as those described previously in^47^. Participants attended one laboratory session lasting approximately ninety minutes. On arrival, participants completed a conditioned learning task, assessing their ability to learn associations between value information and neutral stimuli. They then completed an approach avoidance task, assessing participants’ ability to use the information they learned in the conditioning task to guide behavior. To ensure potential differences in performance on the two tasks were not driven by differences in memory for the learned relationships, participants completed an explicit recall task after undergoing conditioning. Post-experiment all participants were debriefed. Tasks were presented using E-Prime 2.0 on a touch screen Windows PC. An electrocardiogram (ECG) was collected using standard lead II electrode configuration throughout the experiment. To control for any potential differences in cognitive functioning, the Matrix Reasoning and Vocabulary subtests of the Wechsler Abbreviated Scale of Intelligence-Second Edition were administered to all participants (WASI-II)^49^.

### Conditioned value learning

Participants completed a Pavlovian conditioning paradigm where they saw five colored shapes followed by either appetitive, aversive, or neutral reinforcers^33,47^. Appetitive reinforcers consisted of points and a positive image; aversive reinforcers were an unpleasant 95 dB noise and a negative image (Fig. 1). The images were taken from the Open Affective Standardized Image Set (OASIS; Kurdi et al.^50^; Positive Image: I256; Negative Image: I287). During conditioning, participants saw a visual cue (geometric colored shape) that was displayed until a keyboard response was made or 1.5 s had passed. This cue was followed by a delay period of 6 s during which a fixation cross was displayed. The delay was followed by either a corresponding reinforcer or a scrambled neutral image presented for 1.5 s with a probability of 0.8 for the reinforcer and 0.2 for the scrambled neutral image. Each trial was followed by a jittered inter-trial interval of 2.5–5.5 s. A fifth neutral condition consisted of a geometric cue always followed by the neutral scrambled picture. To maintain attention and as a measure of conditioning, participants were asked to press a keyboard response button as soon as they saw the geometric cue. Participants completed 14 trials of each condition for a total of 70 trials. Presentation of each trial was randomized within participants. Across participants, the shape-reinforcer pairings were counterbalanced using a Latin Square design.

To measure conditioned learning, participants were asked to rate how good or bad they thought each neutral shape was prior to and after the conditioning task using a Visual Analogue Scale. Visual Analogue Scale ratings ranged from 0 (Bad) to 100 (Good) (Figure S1). Consistent with previous research, response times from participants’ button press to the neutral shapes were also used as a convergent secondary behavioral measure of learning^33^. These response times were used to model participants’ learning rates during conditioning using a reinforcement learning framework^51^. We derived participant level learning rates using subjects’ response times (RTs) to the cue using participants’ keyboard responses to neutral shapes during the conditioning task. RTs have been shown to be good indicators of conditioning^52,53^, and learning rates represent the speed of integration of recent outcomes^16,25^.

### Use of learned information to guide behavioral choice

After the conditioning task, participants completed a behavioral choice task in which they were asked to use information from the conditioning task to approach or avoid appetitive and aversive stimuli^47^. This task was similar to the conditioned learning task, with the following exceptions. On each trial, participants were presented with the same shapes they had encountered on the previous task. After 1.5 s, a green and a red button appeared on either side of the screen. These buttons remained on screen until participants made a response (Fig. 1). If participants selected the green button, the paired reinforcer was presented. However, if participants selected the red button, a blank screen appeared without any reinforcer. In this manner, selecting the green button represented an approach response and pressing the red button represented an avoidance response. Participants completed 14 trials of each condition for a total of 70 trials. Trial presentation was randomized within participants and the side of the screen where the green and red buttons appeared was counterbalanced across participants.

### Memory of learned information

To ensure that differences in performance on the conditioning task and the behavioral choice task were not a result of participants forgetting the shape-reinforcer pairings, participants also completed an explicit recall task at the end of the experiment^47^. Memory was assessed two different ways. In one block, participants saw each neutral shape and were asked to identify what came after it by selecting one of four choices. In another block, participants were presented with each reinforcer and asked to identify what came before it by selecting one of four choices. Presentation of trials within blocks was randomized, and order of blocks was counterbalanced across participants. Details of the task are shown in Figure S1.

### Physiological measures

As a tertiary convergent measure of learning, we examined autonomic cardiac reactivity during the conditioned learning task. Heart rate was derived from the ECG continuously throughout the study. Results for heart rate are described in inter-beat interval of the heart (IBI). The IBI represents the time in milliseconds between two heart beats, such that as heart rate decreases, IBI increases. The IBI series, derived from ECG, was time sampled at 4 Hz (with interpolation) to yield an equal interval time series. The ECG was measured using a Bionex system (MindWare Technologies LTD, Gahanna, OH). MindWare software was used to visually inspect all physiological data. To examine whether there were differences in autonomic reactivity during anticipation and presentation of reinforcers, IBIs were coded for the six second anticipatory period between cue presentation and reinforcer presentation to assess reactivity in anticipation of the reinforcer. IBIs were also coded for the time period between reinforcer presentation and next cue presentation to assess autonomic reactivity to the reinforcers (4–7 s).

### Statistical analyses

We used hierarchical linear modeling (HLM) techniques to examine participants’ pre- and post-conditioning Visual Analogue Ratings of the neutral shapes by reinforcer conditioning, reaction times to the neutral shapes by reinforcer condition, memory for the shapes by reinforcer condition, behavior on the behavioral choice task, and changes in IBI reactivity to reinforcers during conditioning. All HLM techniques were run using the lmer and glmer functions in the lme4 package in R v3.5.1, the Anova function in the car package was used to examine significance of the fixed effects. The emmeans package to examine simple slopes for interactions in linear models as recommended by Preacher et al.^54^ and estimated marginal effects for predicted response probabilities for interactions in logistic models^55,56^.

To examine whether individuals exhibited different patterns of learning and use behaviors across the conditioned learning and behavioral choice tasks, we used *k-means* clustering methodology^57^. *K-means* takes a data driven approach that allows for identification of latent subgroups across measures of interest – in this case behavioral performance across the two tasks. We opted to use a data driven approach as this allows for identification of potential subgroups of behavioral performance without imposing a priori assumptions about how the behaviors should relate. Our measure of learning during the conditioning task was change in visual analogue scale ratings of the neutral shapes pre- and post-conditioning. Specifically, we calculated unstandardized residualized change scores by subtracting participants’ pre-conditioning rating from their post-conditioning rating. We then regressed the change score onto the pre-conditioning rating to remove baseline variance in ratings^58^. These unstandardized residualized change scores and performance on the behavioral choice task were included as the *k-means* clustering factors. Clusters were run separately for each condition (points, noise, positive image, and negative image). Further methodological and analytic details, including discussion of other potential cluster solutions, are presented in the Supplemental Materials and Table S5.

## Supplementary Information

Supplementary Information.

## Data Availability

Associated data and code is available on the Open Science Framework (OSF; https://osf.io/ns3ke/).
