# Supplementary material for: Children’s value-based decision making
Source: Sci Rep. 2022 Apr 8;12:5953. doi: 10.1038/s41598-022-09894-3 (PMC8993860; doi:10.1038/s41598-022-09894-3)
Supplement: Supplementary file 1 — Supplementary Information. [file 41598_2022_9894_MOESM1_ESM.docx]

Supplemental Materials

Additional Methodological Details

Conditioned Learning Task

Appetitive and aversive images were rated similarly for arousal (Appetitive Image: I256, M_valence_ = 6.49, SD_valence_ = 0.78, M_arousal_ = 5.03, SD_arousal_ = 1.72; Aversive Image: I287, M_valence_ = 1.16, SD_valence_ = 0.42, M_arousal_ = 5.15, SD_arousal_ = 2.34). Points rewards and the aversive noise were accompanied by both visual and auditory presentations: the points were conveyed by an image of a pile of coins and the sound of a cash register, and the aversive noise involved presentation of an alarm bell and the sound of an alarm buzzer. Participants were told they could receive additional money dependent on the number of points earned (up to $5). At the end of the study, participants received the full dollar amount regardless of performance.

Computational Model and Additional Measures of Learning

We used a Rescorla-Wagner rule ^1^ to model learning for each condition. For each trial *t* a prediction error δ(t) was computed as the difference between the actual outcome value R(t) and it’s expected value V(T) on that trial (Eq (1)):

$$\delta\left( t \right)=R\left( t \right)-V(t)$$

Then, the expected value of the next trial V(t + 1) was updated by adding the prediction error δ(t) weighted by a learning rate α (Eq. (2)).

$$V\left( t+1 \right)=V\left( t \right)+ \alpha\delta$$

The outcome value R(t) was set to 1 when a reinforcer was delivered and to 0 when a scrambled picture was displayed. V(t) was initialized to 0. The learning rate, α, represents the speed of integration of recent outcomes ^2,3^.

We derived participant level learning rates using subjects’ response times (RTs) to the cue using participants’ keyboard responses to neutral shapes during the conditioning task. RTs have been shown to be good indicators of conditioning ^4,5^ and correlated with the prediction V(t) estimated by reinforcement learning models ^6^. We derived the prediction V(t) for each participant based on their individual conditioning histories for a range of learning rates (from 0 to 0.5). We then fit trial by trial RTs to a regression model that included the prediction V(t) for each modeled learning and compared regression fits to determine which learning rate best fit participants’ behavior. Across participants the best fit yielded a learning rate of 0.2 which is similar to those utilized in other studies ^7–9^. RTs were in milliseconds and log-transformed to allow analysis across subjects. In line with previous research, we excluded outlier trials with RTs greater than 3 standard deviations from each subjects mean RT ^10^. Using participant level learning rates, we then modeled participant level expected value for each trial to identify participants maximum expected value associated with each neutral shape.

Statistical Analyses

*K-means* clustering is a data-driven approach that allows for the identification of latent subgroups within a data set – in this case latent subgroups of behavior across the two tasks. *K-means* uses a non-hierarchical method of clustering in which each item is assigned to the cluster having the nearest centroid (mean) ^11^. *K-means* analysis initially takes the number of components of the population equal to the final number of clusters, and the final number of clusters is chosen such that the points are mutually farthest apart. Next, it examines each component in the population and assigns it to one of the clusters depending on the minimum distance. The centroid’s position is recalculated every time a components is added to the cluster ad this continues until all components are grouped into the final required number of clusters. The process consists of three steps: 1) partitioning the items into *k* initial clusters; 2) proceeding through the list of items and assigning an item to the cluster whose centroids is nearest, using Euclidean distanced; then recalculating the centroid for the cluster receiving the new item and for the cluster losing the item; 3) repeating step 2 until n more reassignment takes place. We examined clusters using both performance across the entire approach avoidance task and performance on the approach avoidance task for only the first five trials. This allowed us to assess how individuals use learned information with little opportunity for further learning to have occurred. Clusters for adults were more pronounced for analyses using only the first five trials and are those reported in the paper. Cluster using all trials are illustrated in Figures S4 – S6.

As *k-means* analysis requires a pre-identified number of groups, we used the Elbow method to determine the number of clusters that best fit the data ^12^. This method examines the total within-cluster sum of squares (WSS) as a function of the number of clusters, and the number of clusters most appropriate for the data is that at which adding another cluster doesn’t much improve the total WSS. This was assessed using four steps: 1) Computing the clustering algorithm for different numbers of clusters; 2) For each number of clusters, calculate the total WSS; 3) Plot the curve of WSS according to the number of clusters; 4) Using the plot to determine where inclusion of further clusters no longer improves the total WSS (typically the bend of the plot). The Elbow Method identified optimal cluster solutions ranging from three to five clusters (Table S5). Given the four-cluster solution was most consistently implicated as one of the more optimal solutions, all *k-means* analyses were run with 4 clusters. Full WSS statistics and Elbow plots can be found on OSF (<https://osf.io/ns3ke/?view_only=7ad46c4074fd465ebf5fb9f247a22ef5>). Alternative cluster solutions (three and five) are also plotted and available on OSF.

Each participant’s performance on the explicit recall task was measured using: *hit rate* (HR) which refers to the probability of selecting the target; *false alarm rate* (FAR), which refers to the probability of selecting a response which did not match the target. HRs and FARs were combined into a statistic (*d’*) that describes an individual’s sensitivity to targets.

*d'* = z(HR) – z(FAR)

Additional Results

Learning in Conditioning Task

There was an interaction effect between reinforcer type and rating time (pre-/post-conditioning) (χ^2^(4) = 15.28, p = 0.004), suggesting children learned the associated pairings. Examining the simple slopes indicated that children demonstrated increases in ratings of the conditioned stimuli paired with the points (β = 6.00, SE = 3.31, p = 0.07) and positive image (β = 1.59, SE = 3.31, p = 0.63) and decreases in ratings for conditioned stimuli paired with the aversive noise (β = -9.18, SE = 3.31, p = 0.01). However, children demonstrated increases in ratings for the negative image (β = 6.15, SE = 3.31, p = 0.06). We also examined differences in reaction times using hierarchical linear modeling (HLM), including a random intercept for subject and reinforcer condition as a fixed factor. There was no difference in RTs by reinforcer condition (χ^2^(4) = 3.09, p = 0.54). Findings of no difference in RTs by reinforcer type is in line with previous research using a similar task which found differences in valence ratings but not in RTs ^7^.

To further assess learning, we examined heart reactivity to different reinforcer trials during the task utilizing HLM techniques with random effects for reinforcer type nested within subject. For heart rate, time epoch in task was included as a random intercept nested within reinforcer type and interval (anticipation of reinforcer and reinforcer presentation), time epoch, and reinforcer condition (points, appetitive image, aversive noise, negative image, and neutral) were included as fixed factors. Heart rate reactivity was utilized as a tertiary measure as it represents a complex index of learning multiply influenced by a combination of psychological processes, including attention, motivation, and learning ^13–15^*.* In support that children learned the associated pairings there were interactions between interval (anticipation/presentation) and reinforcer (χ^2^(4) = 50.27, p < 0.001), and interval, reinforcer type and time (χ^2^(4) = 20.84, p < 0.001) on IBIs. The interaction between interval and reinforcer type appeared to be driven by the points and aversive noise demonstrating smaller differences in IBIs between anticipation and reinforcer presentation (Table S1). Additionally, examining the simple slopes for the interaction between interval, reinforcer type, and time in task indicated that over the course of the task, IBI reactivity during anticipation decreased for the aversive noise, becoming more similar to reactivity to the reinforcer at the start of the task. Similarly, IBI reactivity for the points and positive images showed less decrease over the course of the task during anticipation suggesting maintenance of IBI reactivity similar to that during reinforcer presentation for the points and positive images (Table S2). Together this is indicative of IBI reactivity during anticipation of the reinforcer, particularly for the points and aversive noise, becoming more similar to that during reinforcer presentation suggestive of learning of paired associations. It is interesting the most pronounced effects for heart rate reactivity appear to be linked to the aversive noise reinforcer, while the points and image reinforcers demonstrate reactivity more similar to that of the neutral image. This suggests the aversive noise may be a particularly salient reinforcer and is likely a result of heart rate being multiply influenced. However, these effects of differential reactivity, in concert with the behavioral findings, suggest children learned the shape-reinforcer pairings, particularly those for the points and aversive noise.

Use of Learned Information in the Behavioral Choice Task

For analyses of approach avoidance behaviors, estimated marginal effects indicated children approached the appetitive reinforcers (Points: M_approach_ = 0.93, SE = 0.02, CI = [0.90, 0.97]; Positive Image: M_approach_ = 0.73, SE = 0.06, CI = [0.62, 0.85]) and avoided the aversive noise more than they approached it (M_approach_ = 0.25, SE = 0.05, CI = [0.14, 0.35]). However, children approached the negative image more than they avoided it (M_approach_ = 0.61, SE = 0.07, CI = [0.48, 0.75]).

Clusters of Performance on the Conditioned Learning Task and Behavioral Choice Tasks Using Alternative Measures of Conditioned Learning

For both analyses run using participant level learning rates and maximum expected value associated with the neutral shape as the measure of learning, we identified four clusters of learning and use that followed the same pattern as that identified with unstandardized residuals (Figures S2 – S3). This provides convergent evidence for this phenomenon across different measures of learning.

**Differences in Clusters for Memory, Age, and General Cognitive Ability.** As reported in the paper, there were no consistent differences in memory, age, and general cognitive ability across the clusters. For age, there was a significant effect of cluster based on learning and use for the points and aversive noise reinforcer for children (Table S3). For the points reinforcer (β = -0.48, SE = 0.15, p = 0.01), post hoc Bonferroni corrected comparisons suggested this was because children in the high learning, low use group were younger than those in the low learning, high use group. For the aversive noise reinforcer, this effect was in the opposite direction (β = 0.46, SE = 0.16, p = 0.03). There were no other significant differences in clusters (ps > 0.07). For general cognitive ability, there was a significant effect of cluster based on learning and use for the positive image reinforcer for children (Table S3). However, none of the post hoc comparisons survived correction (ps > 0.07). None of these differences provide compelling evidence that difference in learning and use behaviors were driven by memory, age, or general cognitive ability.

Follow-up Study with Adults

In Study 2, we examined whether a conditioned learning and behavioral choice task identify similar groups of learners in adults. To do so we aimed to recruit 70 adult participants (see also ^16^). Final recruitment was 74 adults (46 female) between the ages of 18 – 46 years old (M = 19.74; SD = 3.64; Race: 52.7% White Non-Hispanic; 28.4% Asian; 1.4% Black/African American; 4.1% White Hispanic; 4.1% Hispanic; 5.4% Multi-Racial; 4.1% Other). All participants provided written informed consent and received either course credit or a cash payment ($20) for participation. The study was approved by the University of Wisconsin - Madison Institutional Review Board and performed in accordance with all relevant guidelines and regulations. All procedures and tasks were the same as in Study 1. The data was analyzed using the same methods as reported in Study 1, with the exception that a random slope for time was also included for models examining IBI reactivity. Modeling without the random slope produced comparable results and can be found in the analyses on OSF.

Results

**Value Learning as Assessed by the Conditioned Learning Task.** As with children, adults learned the value of the previously neutral stimulus items during conditioning. Adults rated cues paired with appetitive reinforcers more positively after conditioning and those paired with aversive reinforcers more negatively (χ^2^(4) = 58.09, p < 0.001). Again, we found the best fit learning rate across participants was 0.2. Additionally, we found similar effects for heart rate as observed in children. For heart rate, there was a significant interaction between reinforcer condition and interval (χ^2^(4) = 12.18, p = 0.02), indicative of differential heart rate reactivity to the different reinforcers. Including age, gender, and WASI-II score in the models did not change any of the reported effects.

Simple slopes analyses indicated that, as expected, adults demonstrated increases in ratings of the conditioned stimuli paired with the points (β = 7.15, SE = 2.84, p = 0.01) and positive image (β = 8.86, SE = 2.84, p = 0.002) and decreases in ratings for conditioned stimuli paired with the aversive noise (β = -15.54, SE = 2.84, p < 0.001) and negative image (β = -11.16, SE = 2.84, p < 0.001). We also examined differences in reaction times using hierarchical linear modeling (HLM), including a random intercept for subject and reinforcer condition as a fixed factor. There was no difference in RTs by reinforcer condition (χ^2^(4) = 5.07, p = 0.28). Findings of no difference in RTs by reinforcer type is in line with previous research using a similar task which found differences in valence ratings but not in RTs ^7^.

The interaction between reinforcer condition and interval for IBIs was driven by presentation of the reinforcer being associated with increased IBIs (indicative of decreased heart rate) as compared to anticipation of the reinforcer, but this difference was smaller for the aversive noise as compared to the other reinforcers (Table S1). Together this indicates that the aversive noise was associated with greater heart rate responses during anticipation of the reinforcer.

**Value Learning as Assessed by Behavioral Choices.** Adults approached the appetitive reinforcers (Points: M_approach_ = 0.99, SE = 0.003, CI = [0.99, 1.00]; Positive Image: M_approach_ = 0.96, SE = 0.01, CI = [0.93, 0.98]) more than they avoided them and avoided the aversive reinforcers more than they approached them (Aversive Noise: M_approach_ = 0.13, SE = 0.03, CI = [0.06, 0.19]; Negative Image: M_approach_ = 0.20, SE = 0.05, CI = [0.11, 0.29]; χ^2^(4) = 327.85, p < 0.001). Including age, gender, and WASI-II score did not change any of the reported effects.

**Comparing Performance on the Conditioning and Behavioral Choice Tasks.** We examined clusters of behavior using change in pre- and post-conditioning ratings of the neutral shapes and use behaviors on the approach avoidance task as previously done with the child sample. Again, we identified four groups of behavior across the two tasks: a group of individuals with high conditioned learning and high effective use of information; a group of individuals with low conditioned learning and low effective use of information; a group of individuals with high conditioned learning but low effective use of information; and a group of individuals with low conditioned learning and high effective use of information. The four clusters were similar across the different reinforcer conditions (points, positive image, aversive noise, negative image; Figure 2). We ran all cluster analyses using an alternative measure of learning (behavior derived from our computational reinforcement model) and found comparable patterns reported in Figures S2 – S3.

Examination of Alternative Hypotheses

As with children, we found little evidence that an inability to recall the cue reinforcer relationships accounted for performance differences between high and low use groups for high learners (all ps > 0.10 except for clusters based on performance for the points reinforcer; Table S4). There was also no evidence that the relationship between learning and use of value information was associated with participants’ age, gender, or general cognitive ability (ps > 0.10).

However, there were significant differences by cluster for sensitivity (Table S3). For all reinforcers except the points reinforcer these effects were not driven by differences in the sensitivity index between the high learning, high use group and high learning, low use group (Table S4). This suggests differences in memory for the relationships were not driving the observed separation between learning and use. There were no other significant differences in clusters. None of these differences provide compelling evidence that difference in learning and use behaviors were driven by memory, age, or general cognitive ability.

**Table S1**

Simple slopes for interaction between reinforcer condition and interval for IBIs

|  | Child | | Adult | |
| --- | --- | --- | --- | --- |
| Reinforcer Condition | β (SE) | p | β (SE) | p |
| Neutral | -15.90 (4.70) | 0.001 | -19.01 (4.66) | < 0.001 |
| Points | -17.20 (4.65) | < 0.001 | -19.30 (4.87) | < 0.001 |
| Positive Image | -18.20 (4.61) | < 0.001 | -11.64 (4.64) | 0.01 |
| Aversive Noise | 15.70 (4.77) | 0.001 | -8.15 (4.70) | 0.08 |
| Negative Image | -28.70 (4.65) | < 0.001 | -29.37 (4.69) | < 0.001 |

*Note*. Slope represents anticipation - stimulus

**Table S2**

Simple slopes for interaction between reinforcer condition, interval, and time for IBIs for children

| Reinforcer Condition | Interval | β (SE) | p |
| --- | --- | --- | --- |
| Neutral | Anticipation | -0.25 (0.04) | < 0.001 |
|  | Stimulus | -0.33 (0.04) | < 0.001 |
| Points | Anticipation | -0.14 (0.04) | < 0.001 |
|  | Stimulus | -0.29 (0.04) | < 0.001 |
| Positive Image | Anticipation | -0.20 (0.04) | < 0.001 |
|  | Stimulus | -0.27 (0.04) | < 0.001 |
| Aversive Noise | Anticipation | -0.27 (0.04) | < 0.001 |
|  | Stimulus | -0.08 (0.04) | 0.06 |
| Negative Image | Anticipation | -0.26 (0.04) | < 0.001 |
|  | Stimulus | -0.37 (0.04) | < 0.001 |

**Table S3**

Effects for age, general cognitive ability, memory, and gender by cluster group

|  |  | Child | | Adults | |
| --- | --- | --- | --- | --- | --- |
| Variable | Reinforcer Condition | F (df) | p | F (df) | p |
| Age | Points | 4.82 (3,67) | 0.004 | 1.24 (3,69) | 0.30 |
|  | Positive Image | 1.32 (3,67) | 0.28 | 0.88 (3,69) | 0.46 |
|  | Aversive Noise | 2.92 (3,67) | 0.04 | 2.70 (3,69) | 0.05 |
|  | Negative Image | 1.86 (3,67) | 0.14 | 1.54 (3,69) | 0.21 |
| WASI-II Score | Points | 0.04 (3,65) | 0.99 | 1.52 (3,69) | 0.22 |
|  | Positive Image | 2.89 (3,65) | 0.04 | 0.80 (3,69) | 0.50 |
|  | Aversive Noise | 1.13 (3,65) | 0.34 | 1.42 (3,69) | 0.24 |
|  | Negative Image | 0.48 (3,65) | 0.70 | 0.38 (3,69) | 0.77 |
| Memory (Sensitivity) | Points | 1.86 (3,66) | 0.15 | 20.37 (3,70) | < 0.001 |
|  | Positive Image | 0.53 (3,66) | 0.66 | 10.77 (3,70) | < 0.001 |
|  | Aversive Noise | 0.96 (3,66) | 0.42 | 13.28 (3,70) | < 0.001 |
|  | Negative Image | 0.86 (3,66) | 0.47 | 11.75 (3,70) | < 0.001 |
|  | |  |  | χ^2^ (df) | p |
| Gender | Points | 5.59 (3) | 0.13 | 2.31 (3) | 0.51 |
|  | Positive Image | 2.12 (3) | 0.55 | 4.60 (3) | 0.20 |
|  | Aversive Noise | 0.31 (3) | 0.96 | 1.82 (3) | 0.61 |
|  | Negative Image | 3.98 (3) | 0.26 | 1.13 (3) | 0.77 |

Note. For age and general cognitive ability (measure using WASI-II), models were run as ANOVAs including cluster as a between subject factor with age and WASI-II score as the outcome variables. For gender, the model was run as logistic regression with including cluster as a predictor variable and gender as a binary outcome.

**Table S4**

Bonferroni corrected comparison for sensitivity index by cluster for adults

| Reinforcer Type | Comparison | β(SE) | t | p |
| --- | --- | --- | --- | --- |
| Points | High learning, high use – High learning, low use | 3.80 (0.63) | 6.08 | < 0.001 |
|  | High learning, high use – Low learning, high use | 2.22 (0.35) | 6.42 | < 0.001 |
|  | High learning, high use – Low learning, low use | 3.00 (1.02) | 2.96 | 0.03 |
|  | High learning, low use – Low learning, high use | -1.58 (0.61) | -2.60 | 0.07 |
|  | High learning, low use – Low learning, low use | -0.80 (1.13) | -0.70 | 0.99 |
|  | Low learning, high use – low learning, low use | 0.78 (1.00) | 0.78 | 0.99 |
| Positive Image | High learning, high use – High learning, low use | 1.84 (0.68) | 2.59 | 0.05 |
|  | High learning, high use – Low learning, high use | 2.32 (0.41) | 5.62 | < 0.001 |
|  | High learning, high use – Low learning, low use | 1.28 (0.72) | 1.77 | 0.49 |
|  | High learning, low use – Low learning, high use | 0.48 (0.67) | 0.72 | 0.99 |
|  | High learning, low use – Low learning, low use | -0.56 (0.89) | -0.63 | 0.99 |
|  | Low learning, high use – low learning, low use | -1.04 (0.71) | -1.47 | 0.88 |
| Aversive Noise | High learning, high use – High learning, low use | 1.48 (0.61) | 2.42 | 0.11 |
|  | High learning, high use – Low learning, high use | 2.38 (0.47) | 5.05 | < 0.001 |
|  | High learning, high use – Low learning, low use | 2.73 (0.47) | 5.85 | < 0.001 |
|  | High learning, low use – Low learning, high use | 0.90 (0.60) | 1.49 | 0.85 |
|  | High learning, low use – Low learning, low use | 1.25 (0.60) | 2.08 | 0.25 |
|  | Low learning, high use – low learning, low use | 0.35 (0.46) | 0.78 | 0.99 |
| Negative Image | High learning, high use – High learning, low use | 0.96 (0.58) | 1.67 | 0.60 |
|  | High learning, high use – Low learning, high use | 2.69 (0.49) | 5.49 | < 0.001 |
|  | High learning, high use – Low learning, low use | 2.22 (0.52) | 4.26 | < 0.001 |
|  | High learning, low use – Low learning, high use | 1.73 (0.53) | 3.25 | 0.01 |
|  | High learning, low use – Low learning, low use | 1.26 (0.56) | 2.24 | 0.17 |
|  | Low learning, high use – low learning, low use | -0.48 (0.48) | -1.00 | 0.99 |

Table S5

*Optimal Cluster Solutions for Different Reinforcer Types and Learning Outcomes Based on Elbow Method*

|  | Reinforcer Type | Children | Adults |
| --- | --- | --- | --- |
| VAS Ratings – Approach Avoid First Five Trials | Points | 3 or 4 | 3, 4, 5, or 6 |
|  | Positive Image | 3, 4, or 5 | 3, 4, or 5 |
|  | Aversive Noise | 3, 4, or 5 | 3, 4, or 5 |
|  | Negative Image | 4 | 3 |
| VAS Ratings – Approach Avoid All Trials | Points | 3, 4, or 5 | 3, 4, or 5 |
|  | Positive Image | 3, 4, or 5 | 3 |
|  | Aversive Noise | 3, 4, or 5 | 3, 4, or 5 |
|  | Negative Image | 4 | 3, 4, or 5 |
| Learning Rates – Approach Avoid First Five Trials | Points | 4 | 3 or 4 |
|  | Positive Image | 3 or 4 | 3, 4, or 5 |
|  | Aversive Noise | 4 | 3, 4, or 5 |
|  | Negative Image | 3, 4, 5, or 6 | 3, 4, or 5 |
| Learning Rates – Approach Avoid All Trials | Points | 3, 4, or 5 | 3, 4, or 5 |
|  | Positive Image | 3, 4, or 5 | 3, 4, or 5 |
|  | Aversive Noise | 4 | 5 |
|  | Negative Image | 5 | 3, 4, or 5 |
| Max Value – Approach Avoid First Five Trials | Points | 3, 4, or 5 | 3 - 7 |
|  | Positive Image | 4 | 3 - 6 |
|  | Aversive Noise | 3, 4, or 5 | 3 - 7 |
|  | Negative Image | 3, 4, or 5 | 4 |
| Max Value – Approach Avoid All Trials | Points | 3, 4, or 5 | 4 |
|  | Positive Image | 4 | 3, 4, or 5 |
|  | Aversive Noise | 3, 4, or 5 | 3 - 6 |
|  | Negative Image | 3, 4, or 5 | 3, 4, or 5 |

Good

Bad

How good or bad is this?


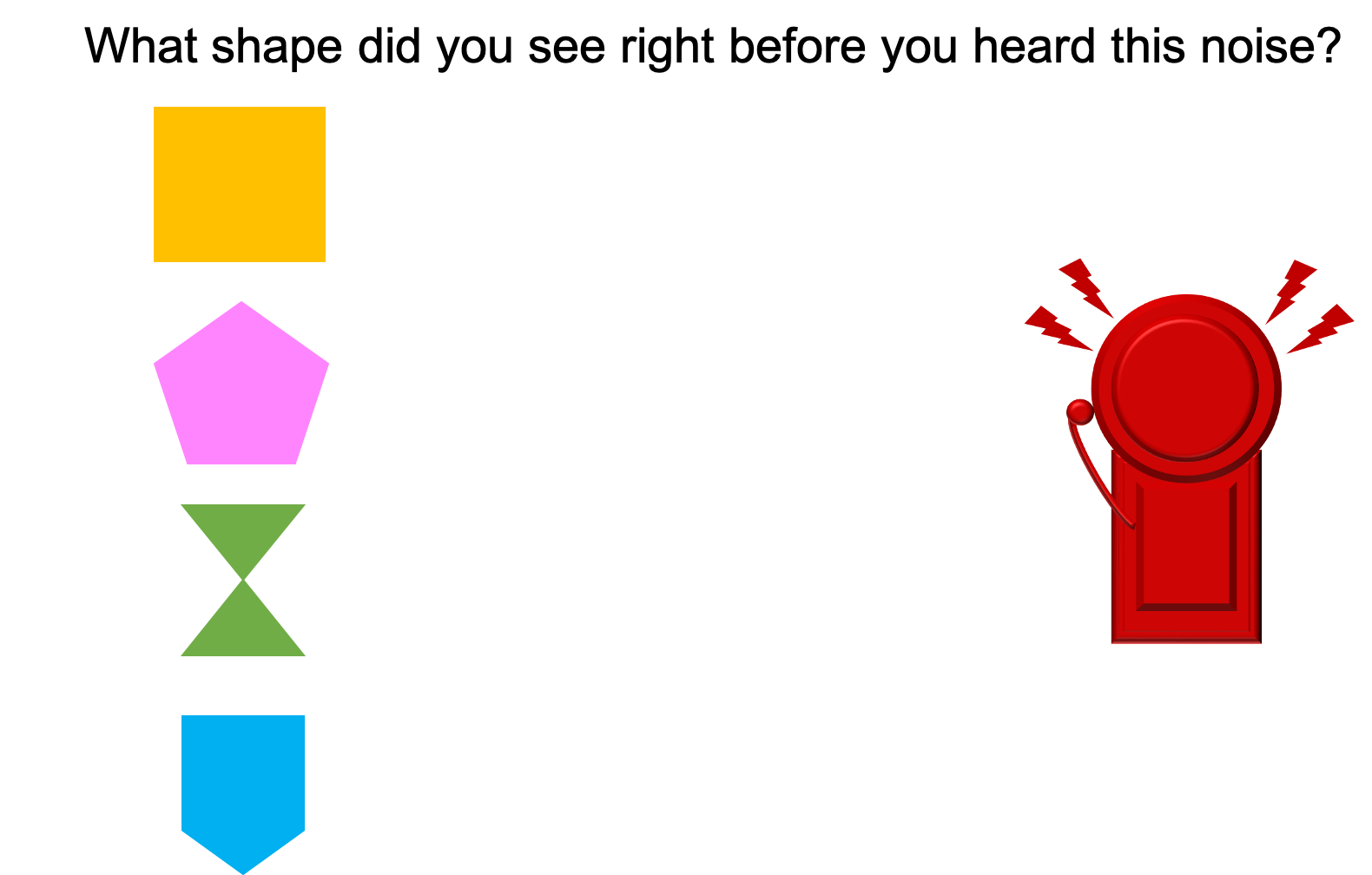


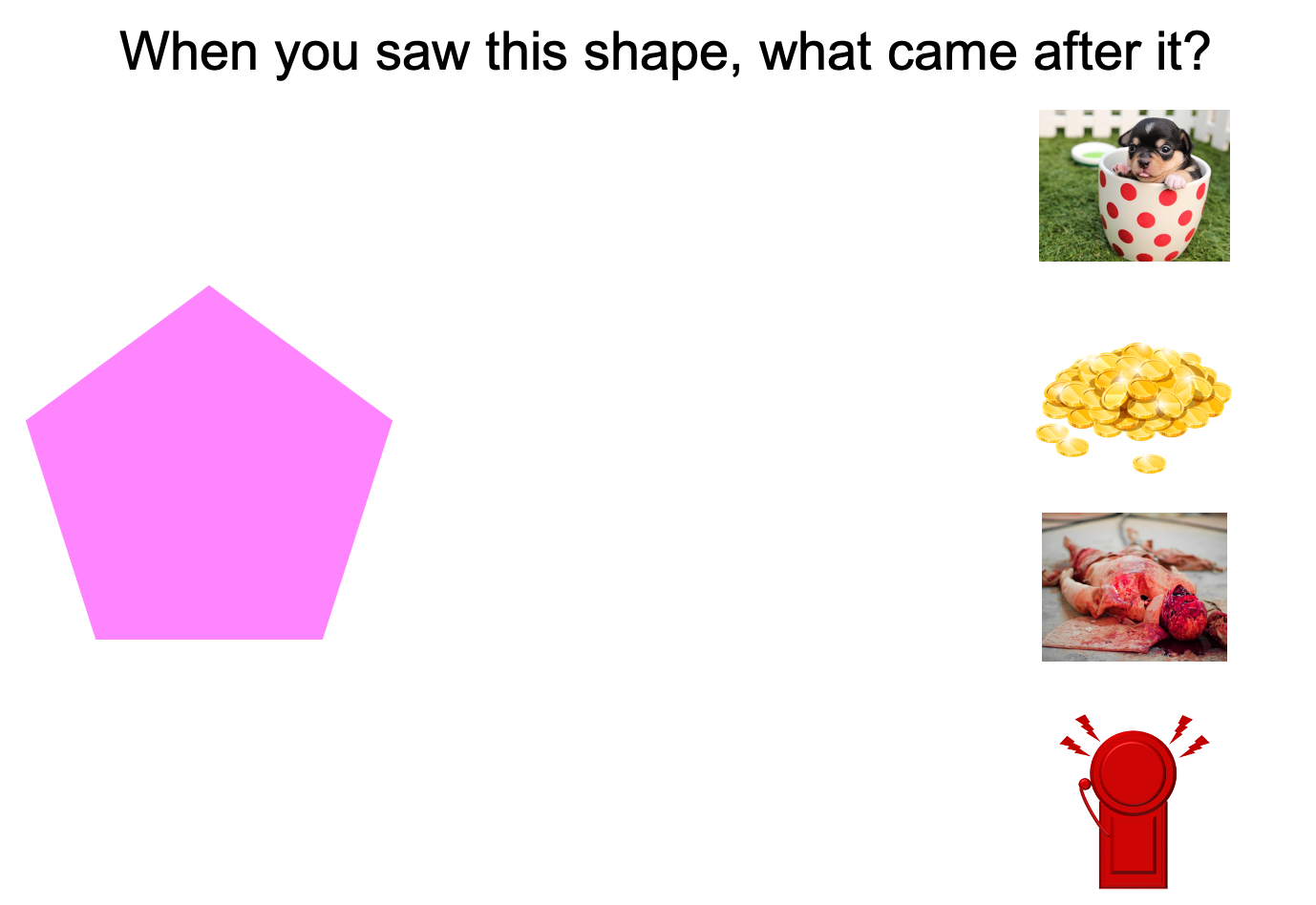


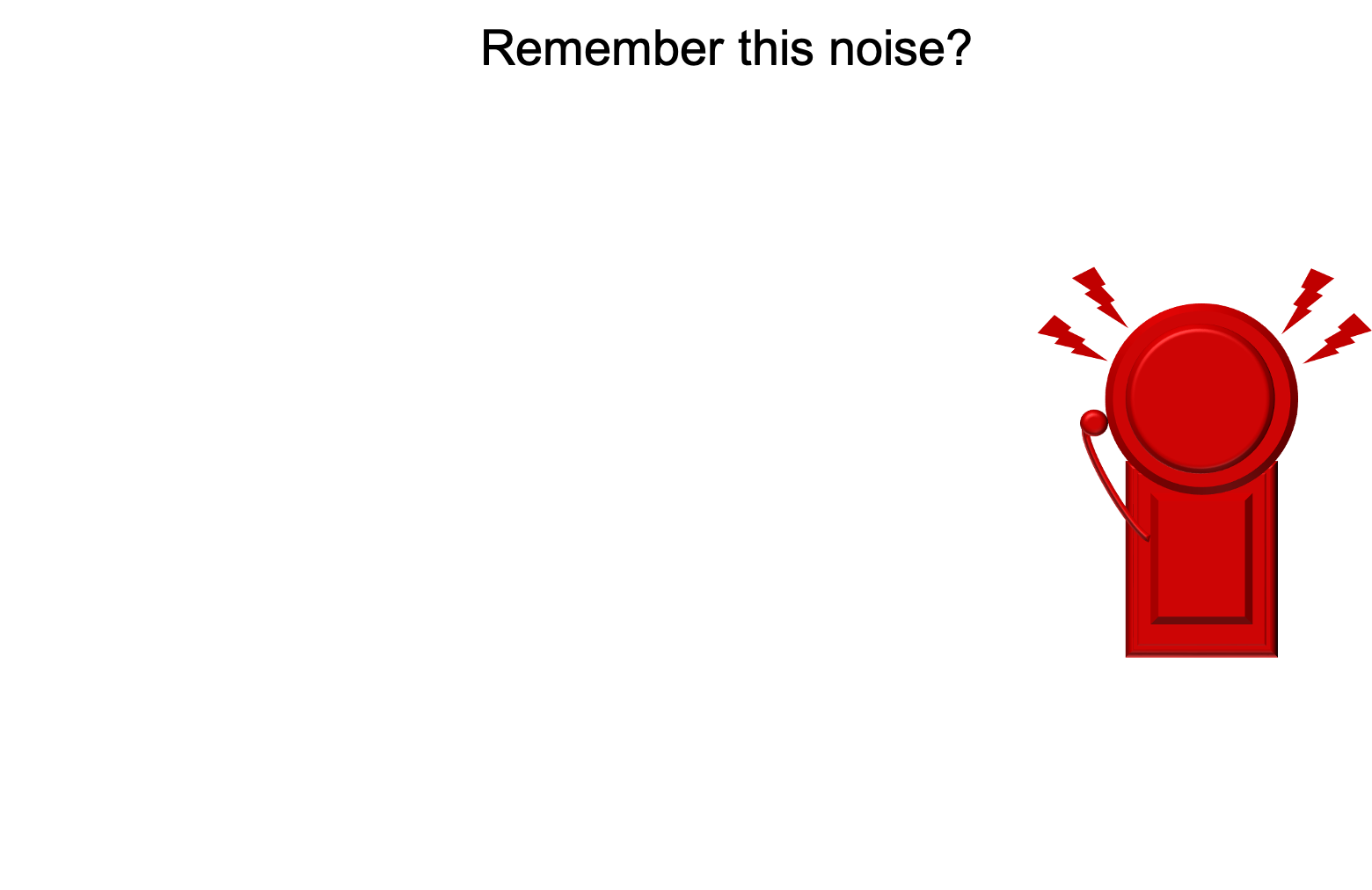


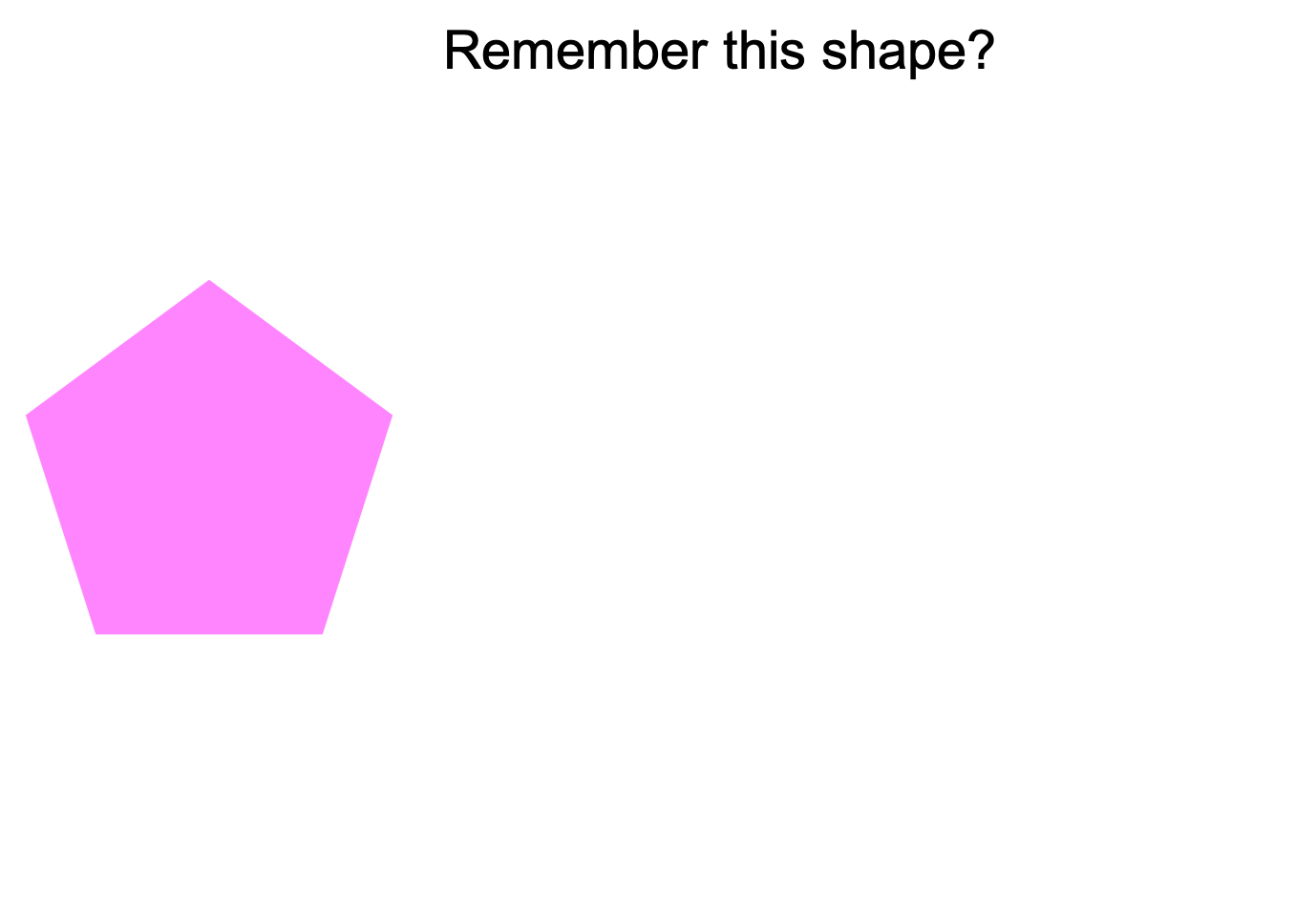


**Figure S1**

*Procedure for Pre- and Post-conditioning Ratings and Explicit Recall Task*

A)

B)

1.5s

1.5s


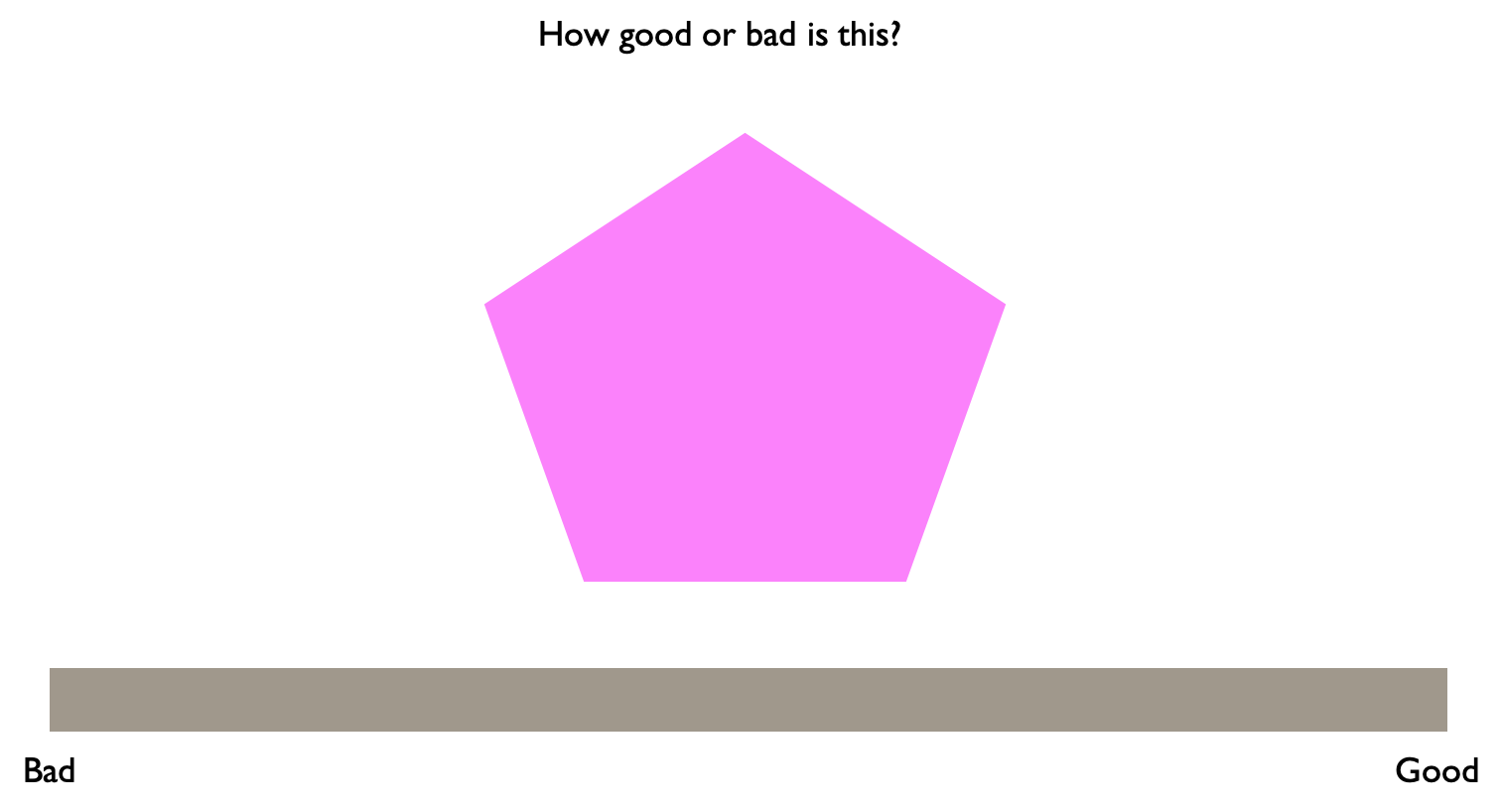


*Note.* A) Procedure for pre- and post-conditioning ratings of neutral shapes using Visual Analogue Scale (VAS) ranging from bad (0) to good (100). Participants were asked to respond to “How good or bad is this” by clicking on the gray scale bar. B) Procedure for explicit recall task. This task consisted of two blocks, counterbalanced across participants. In one block, participants saw the shape for 1.5 seconds and were asked to identify what came after the shape. In the other block, participants saw the reinforcer and were asked to identify what shape came before it.

**Figure S2**

*Clusters based on learning rate modeled using reaction times and use for each reinforcer condition.*


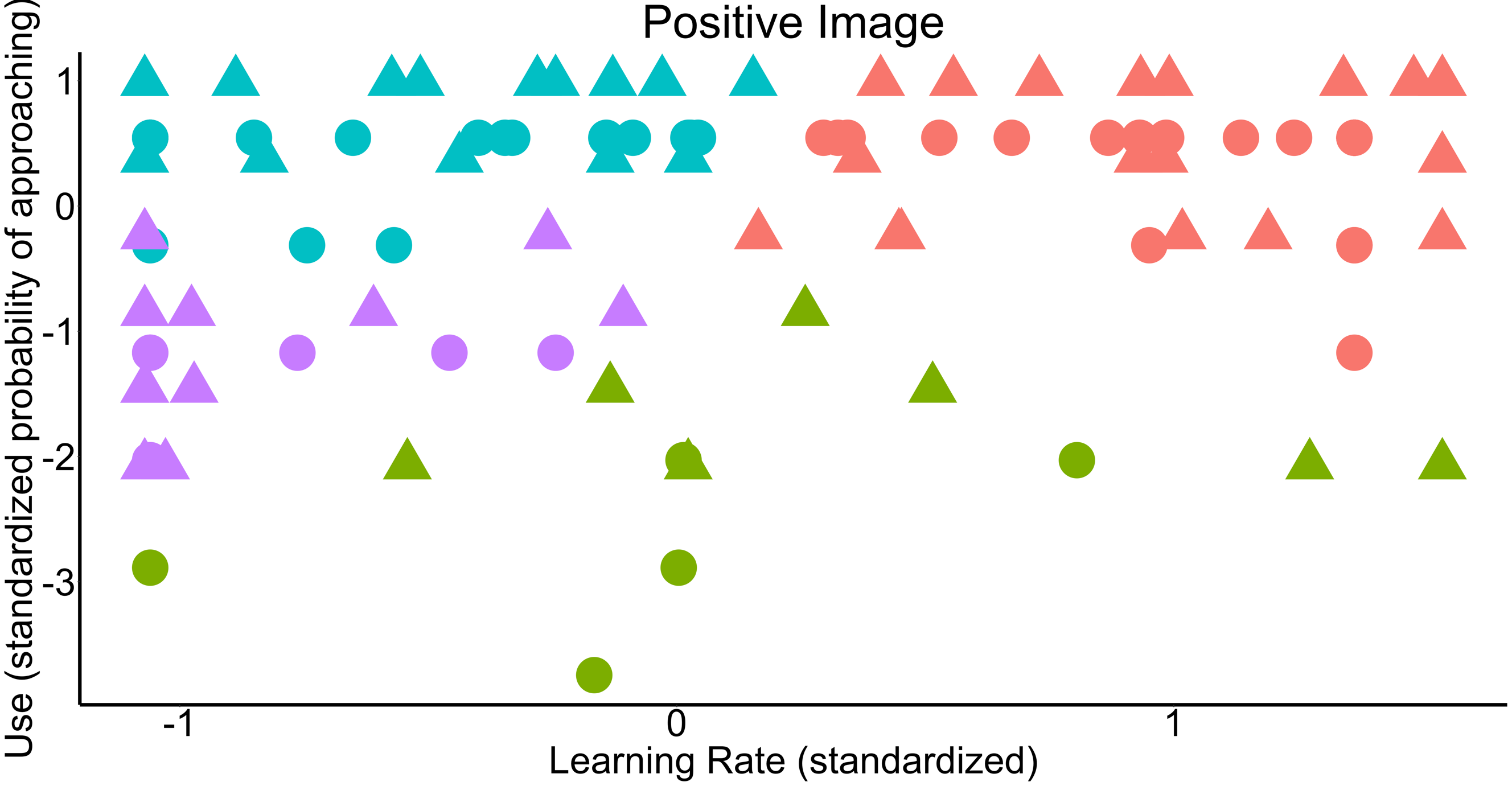


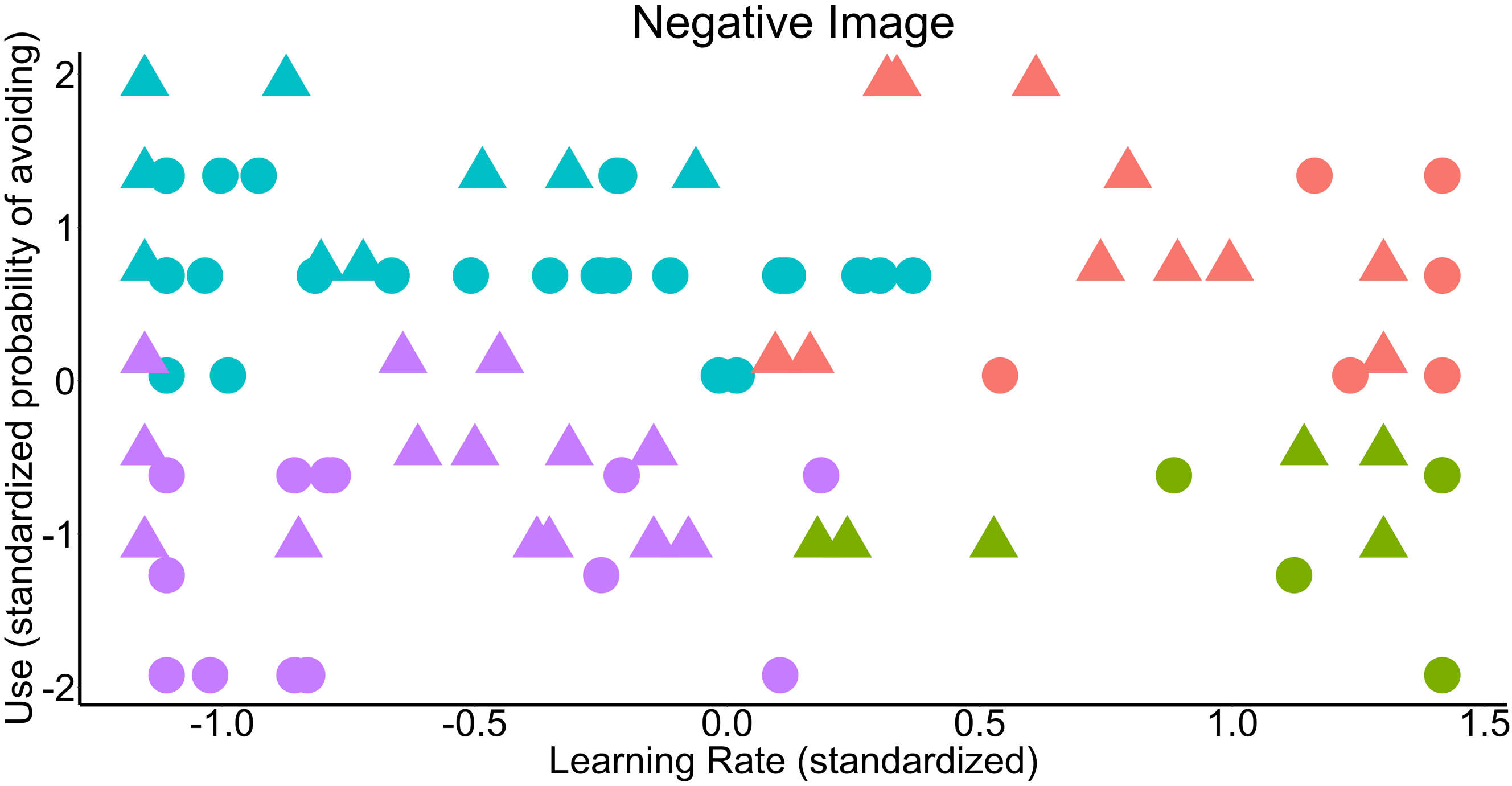


Appetite Reinforcers

Aversive Reinforcers


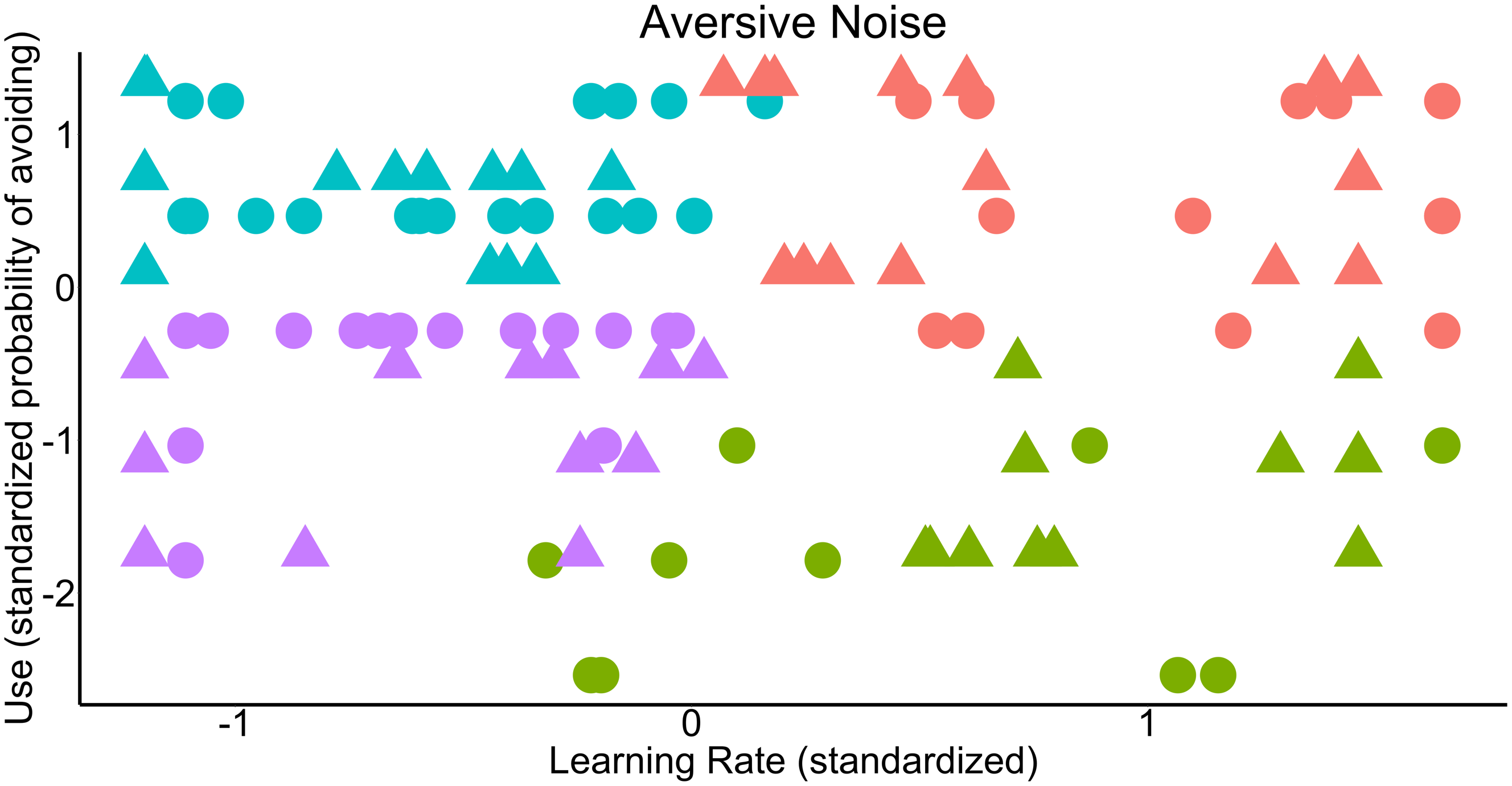


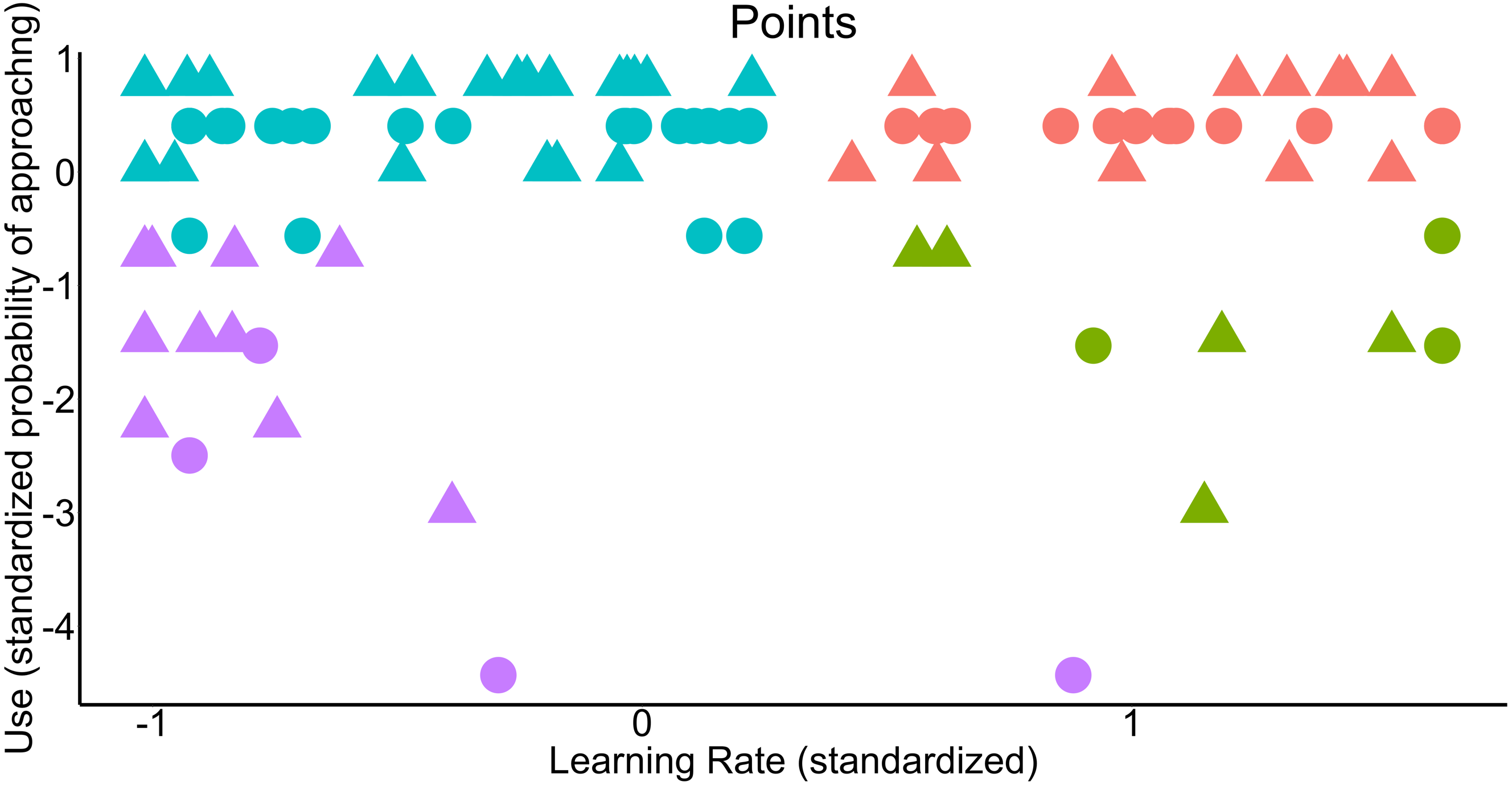


Adults

Children

Low Learning, High Use

High Learning, High Use

High Learning, Low Use

Low Learning, Low Use

**Figure S3**

*Clusters based on maximum expected value modeled using reaction times and use for each reinforcer condition.*

Appetite Reinforcers

Aversive Reinforcers


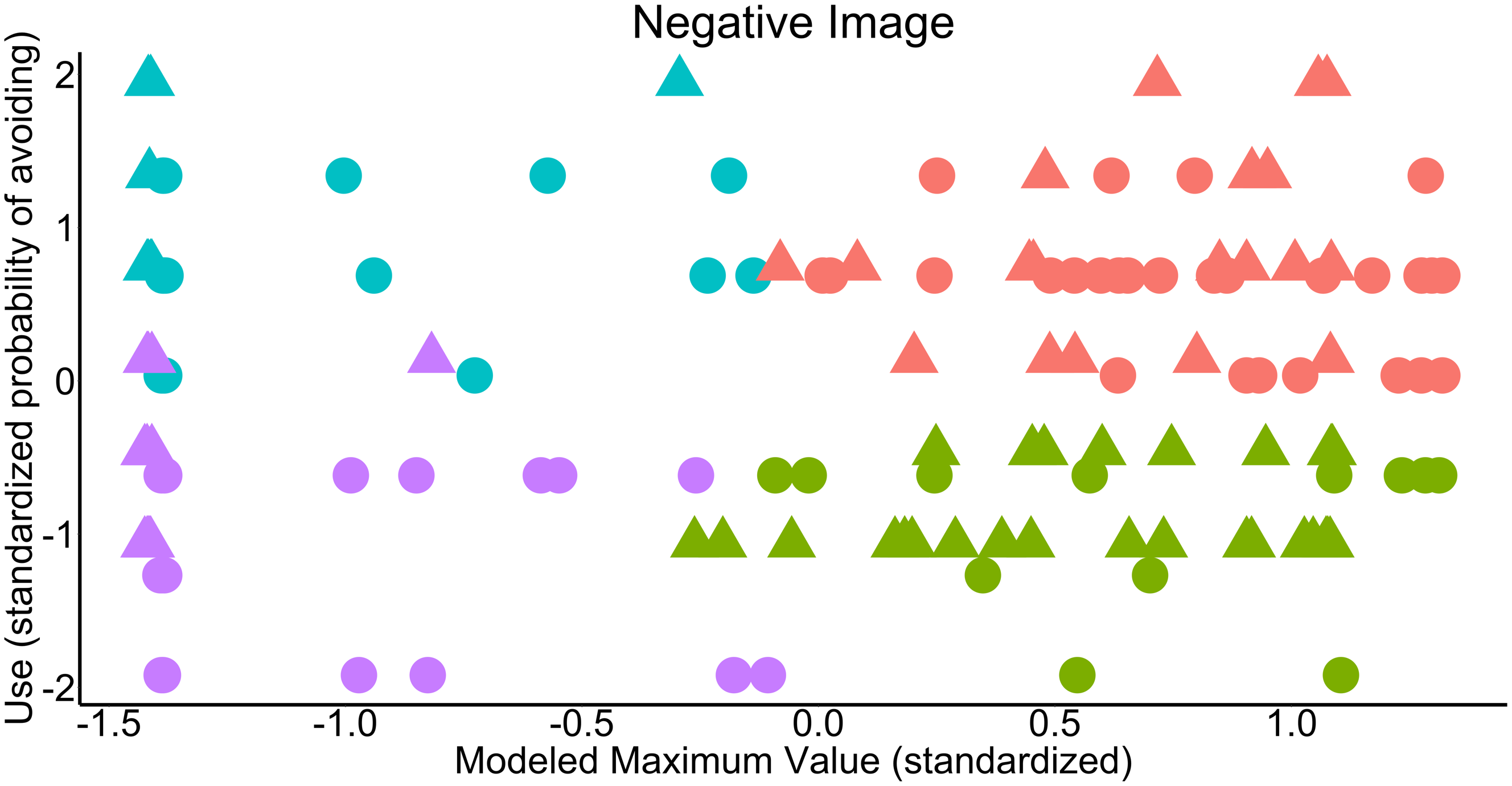


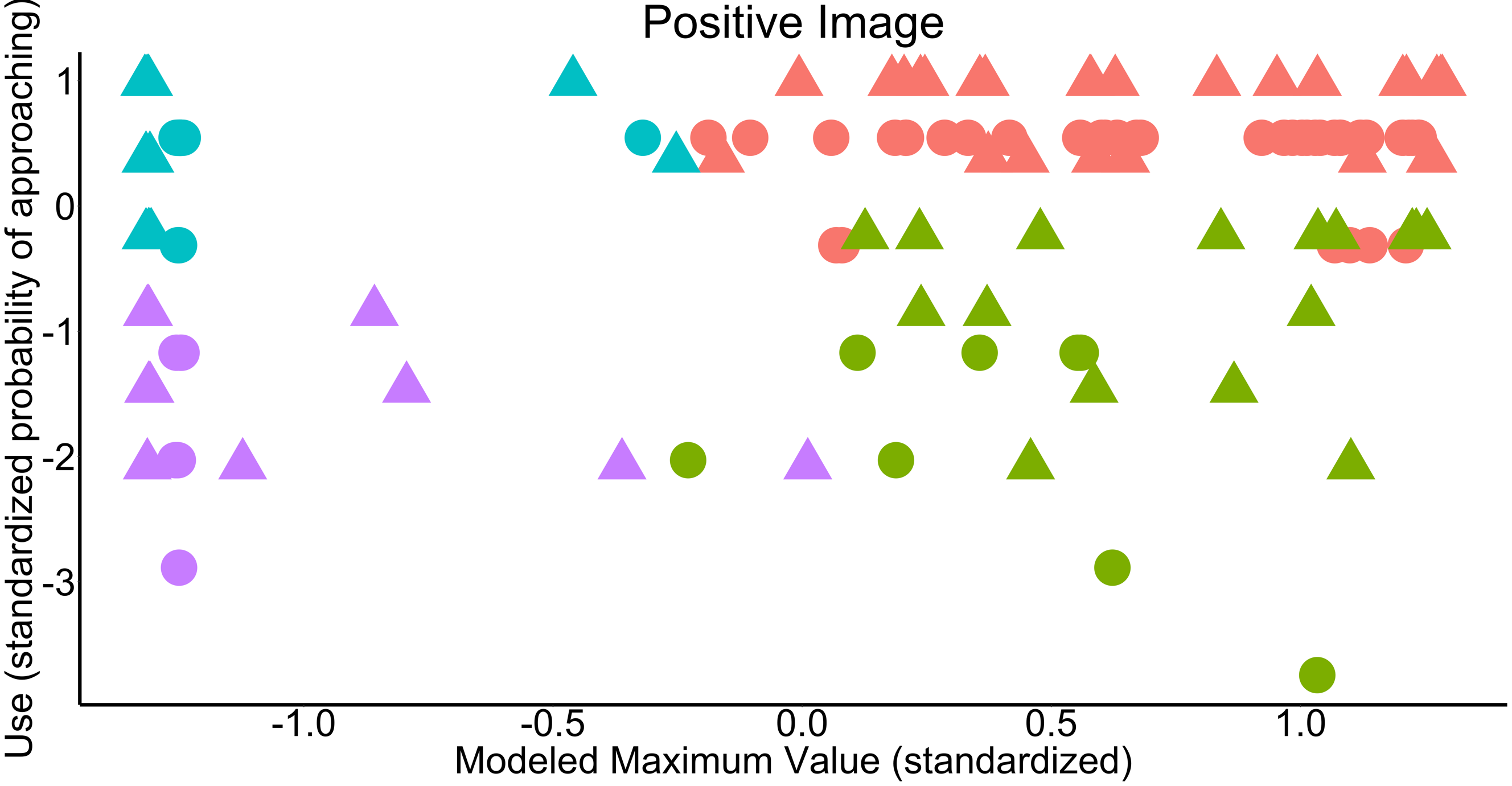


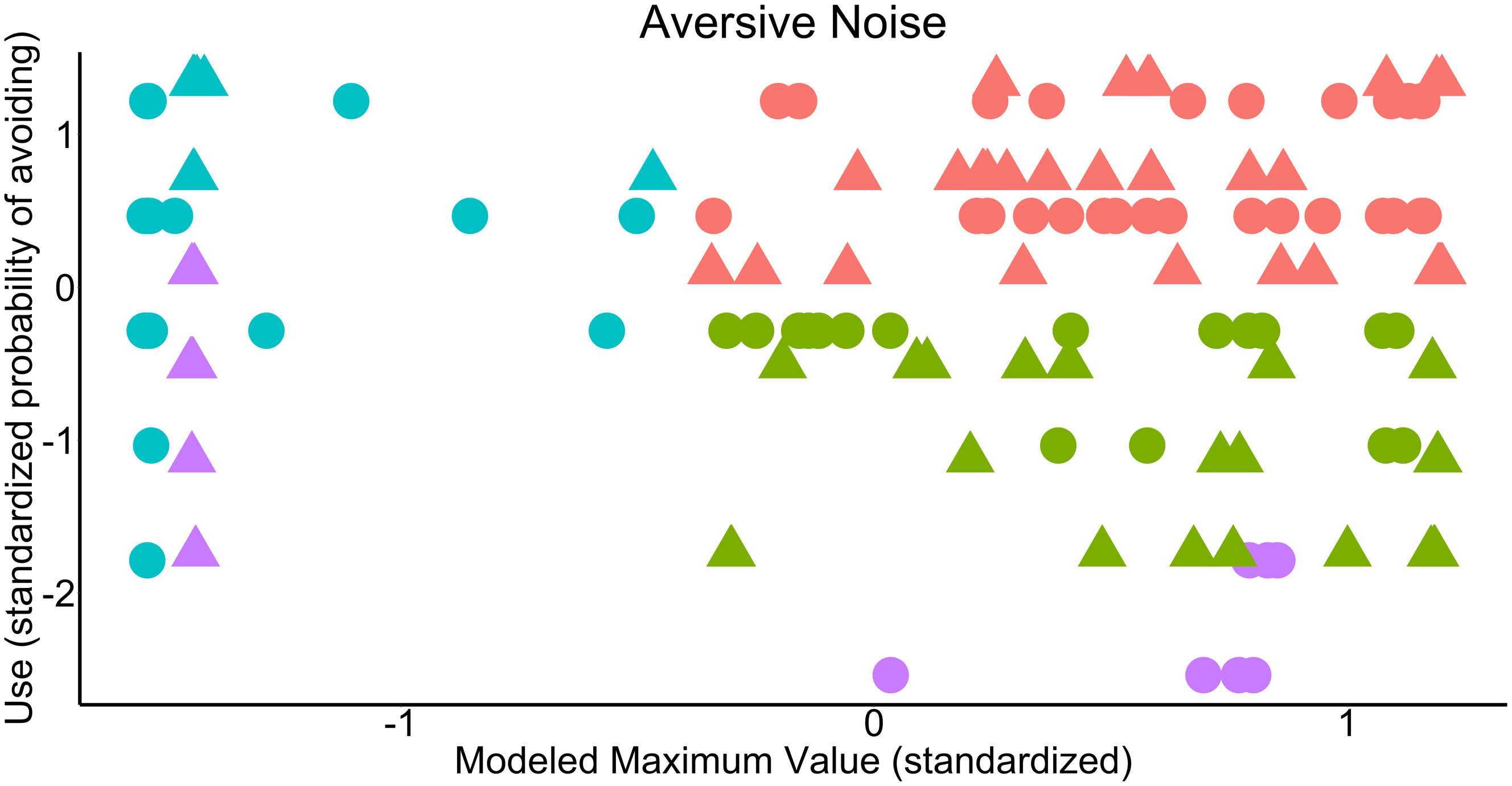


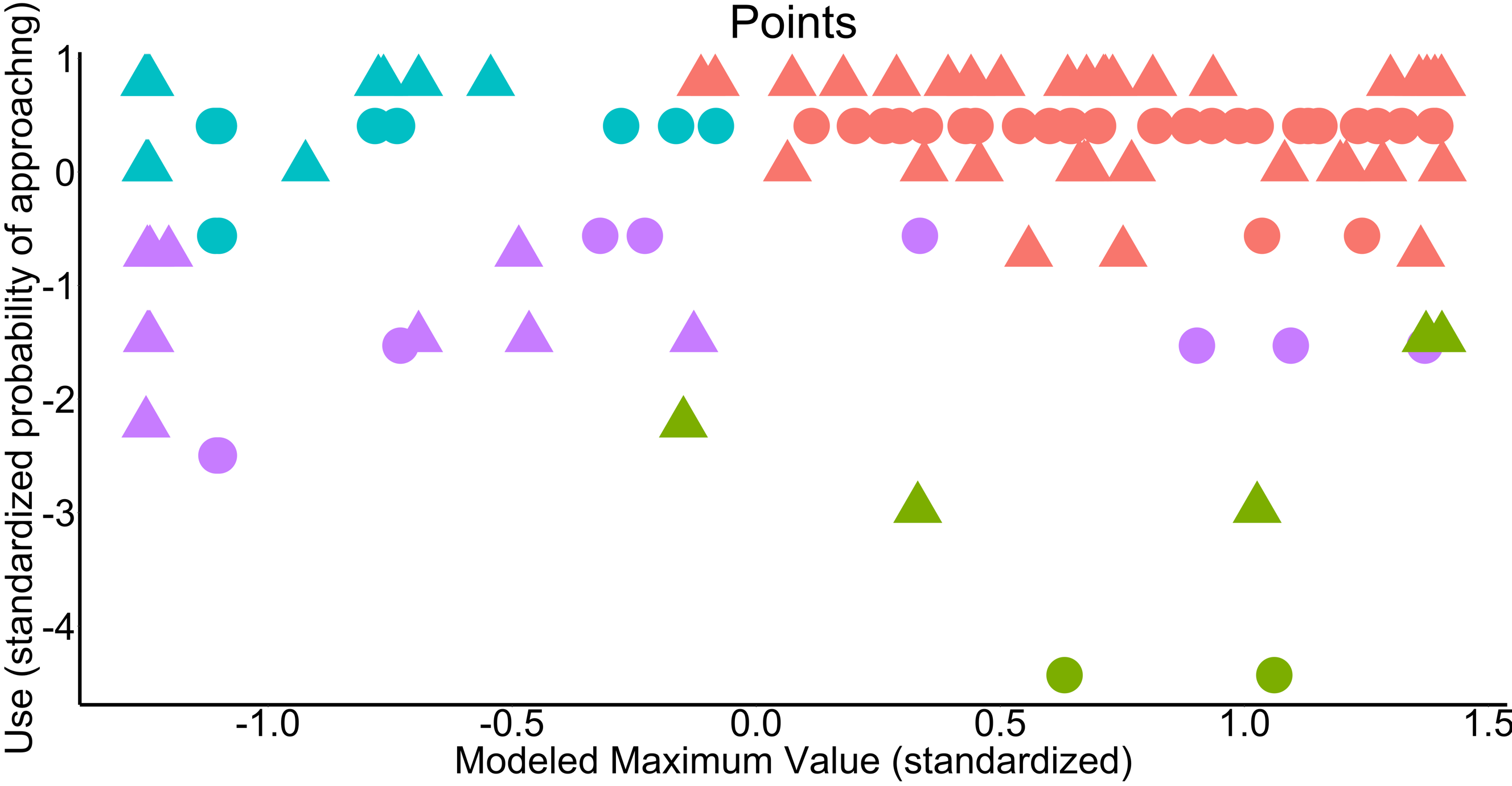


Children

Adults

Low Value, High Use

High Value, Low Use

High Value, High Use

Low Value, Low Use

Children

Adults

**Figure S4**

*Clusters based on unstandardized residualized change scores for VAS ratings and use averaged across full trials for each reinforcer condition. All axes are standardized.*

High Learning, High Use

High Learning, Low Use


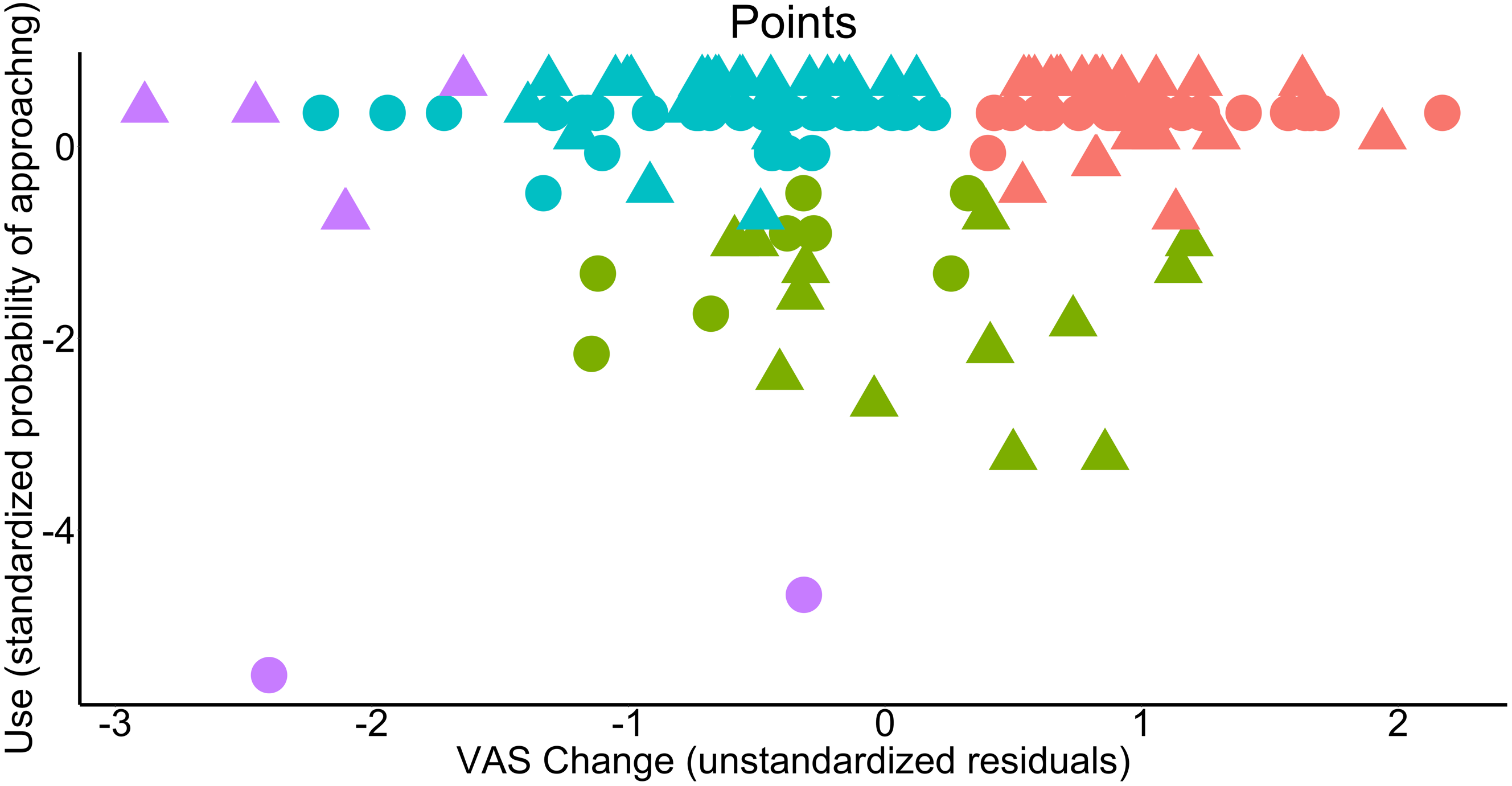


Appetite Reinforcers

Aversive Reinforcers

Low Learning, High Use

Low Learning, Low Use


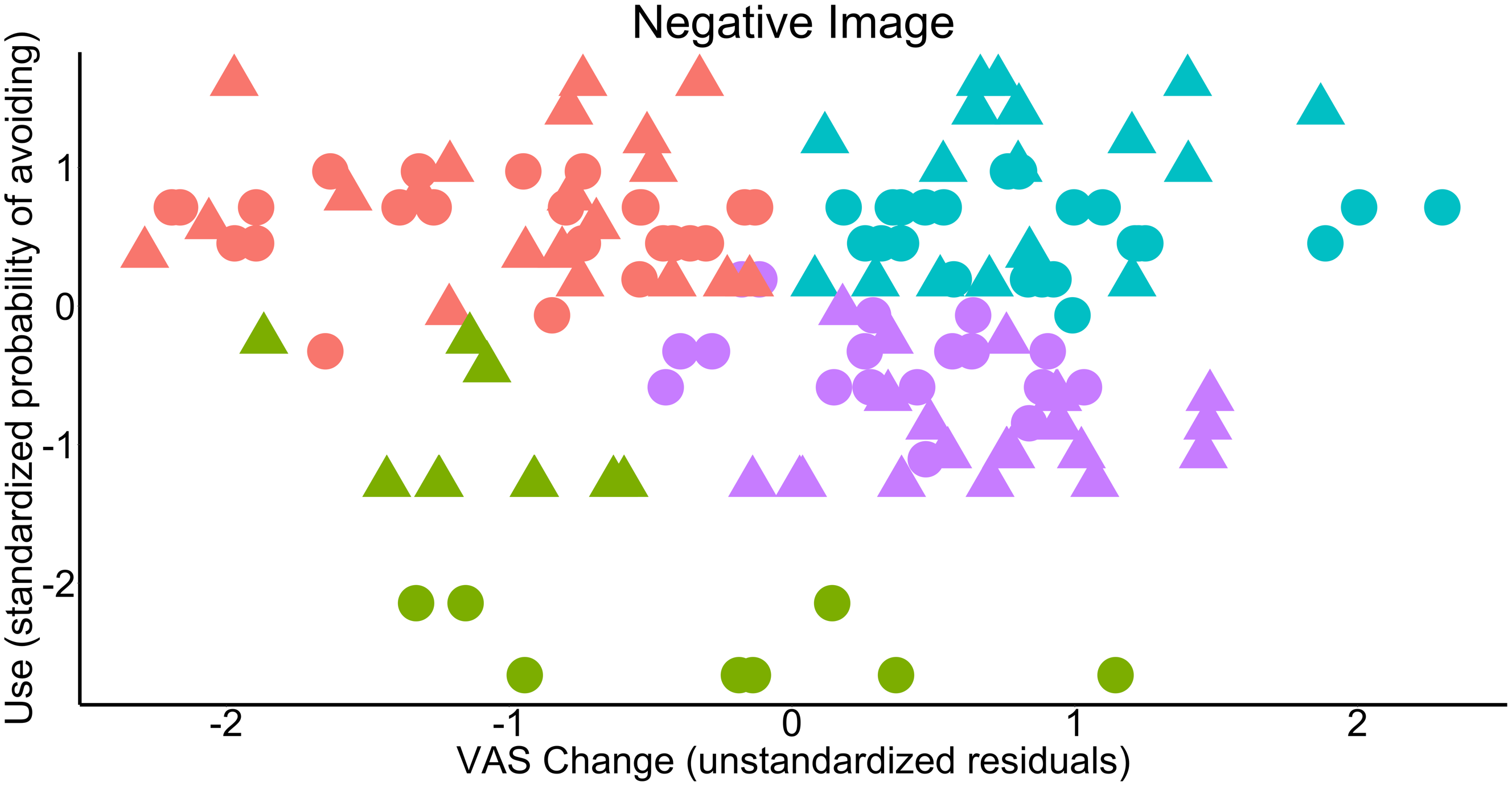


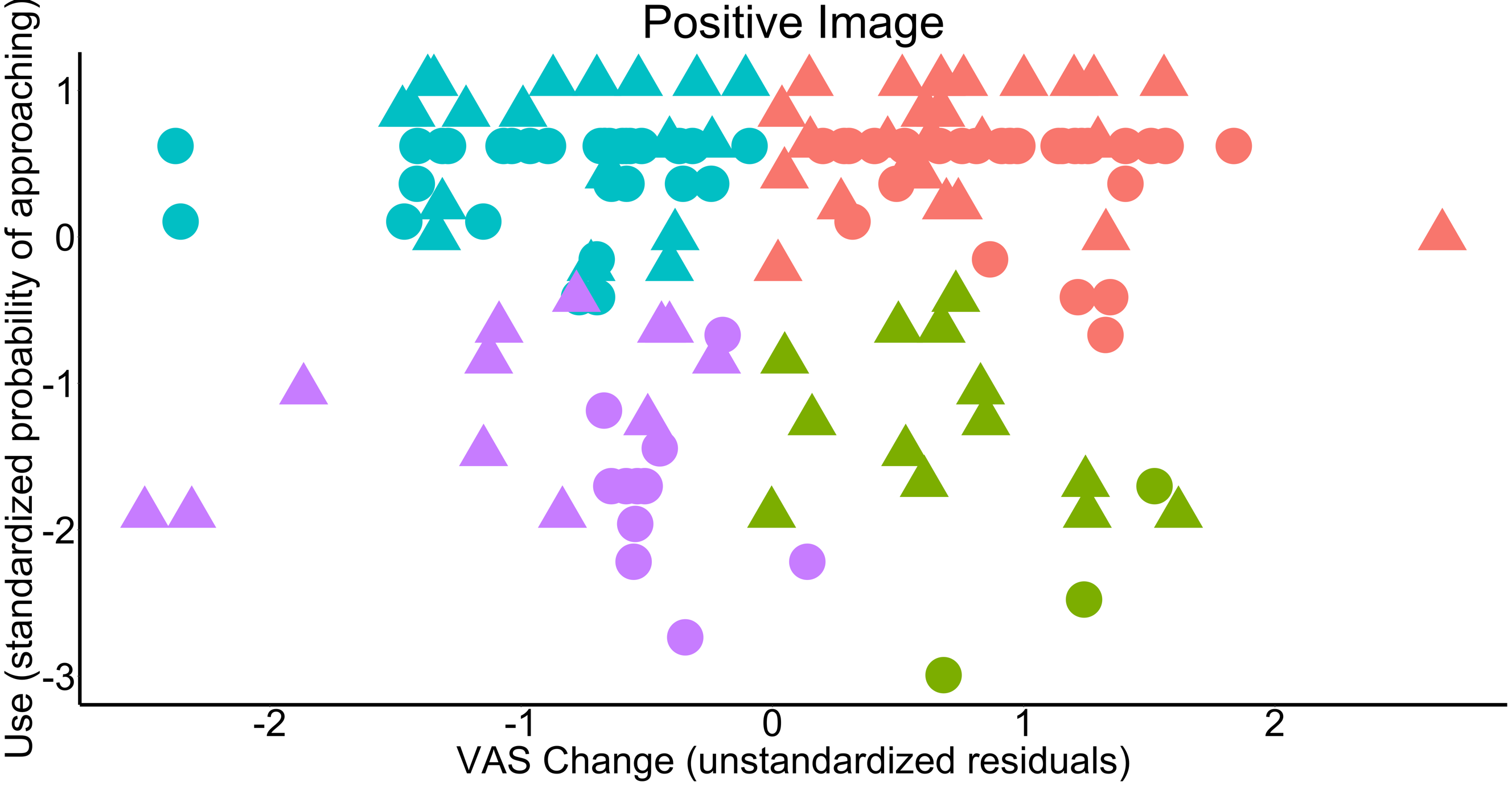


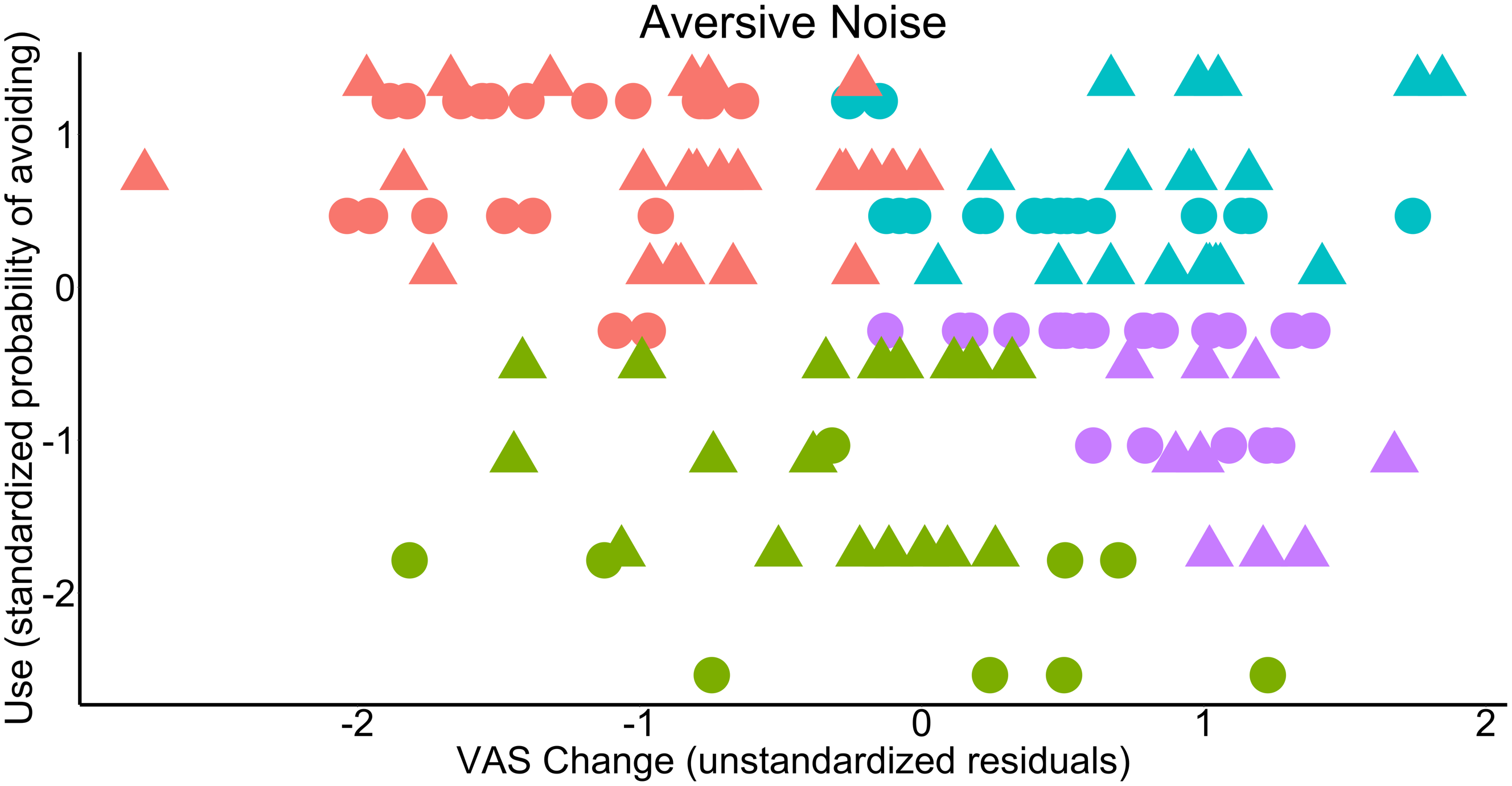


Children

Adults

**Figure S5**

*Clusters based on learning rate modeled using reaction times and use averaged across full trials for each reinforcer condition.*


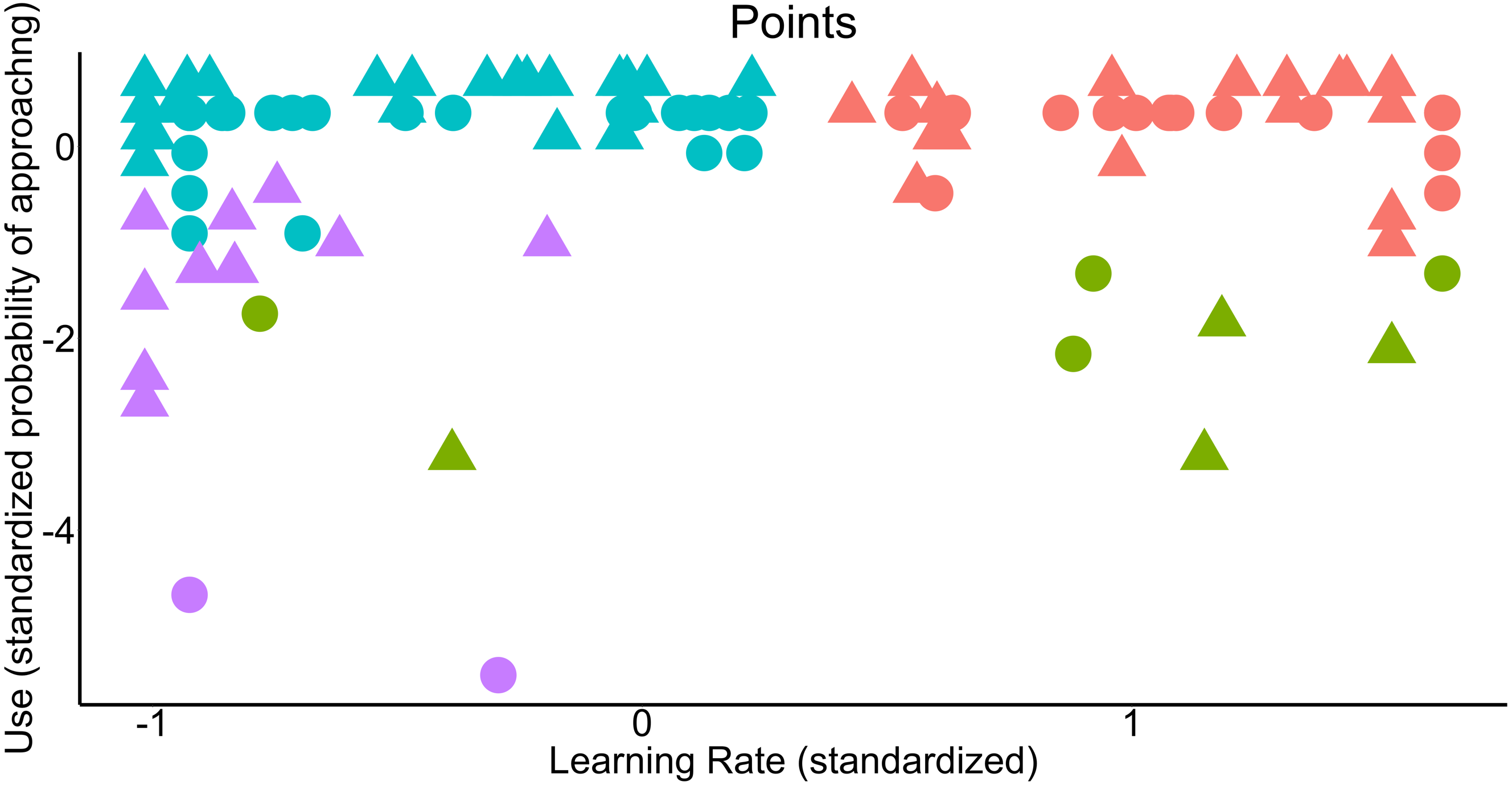


Appetite Reinforcers

Aversive Reinforcers

Low Learning Rate, Low Use

Low Learning Rate, High Use

High Learning Rate, Low Use

High Learning Rate, High Use


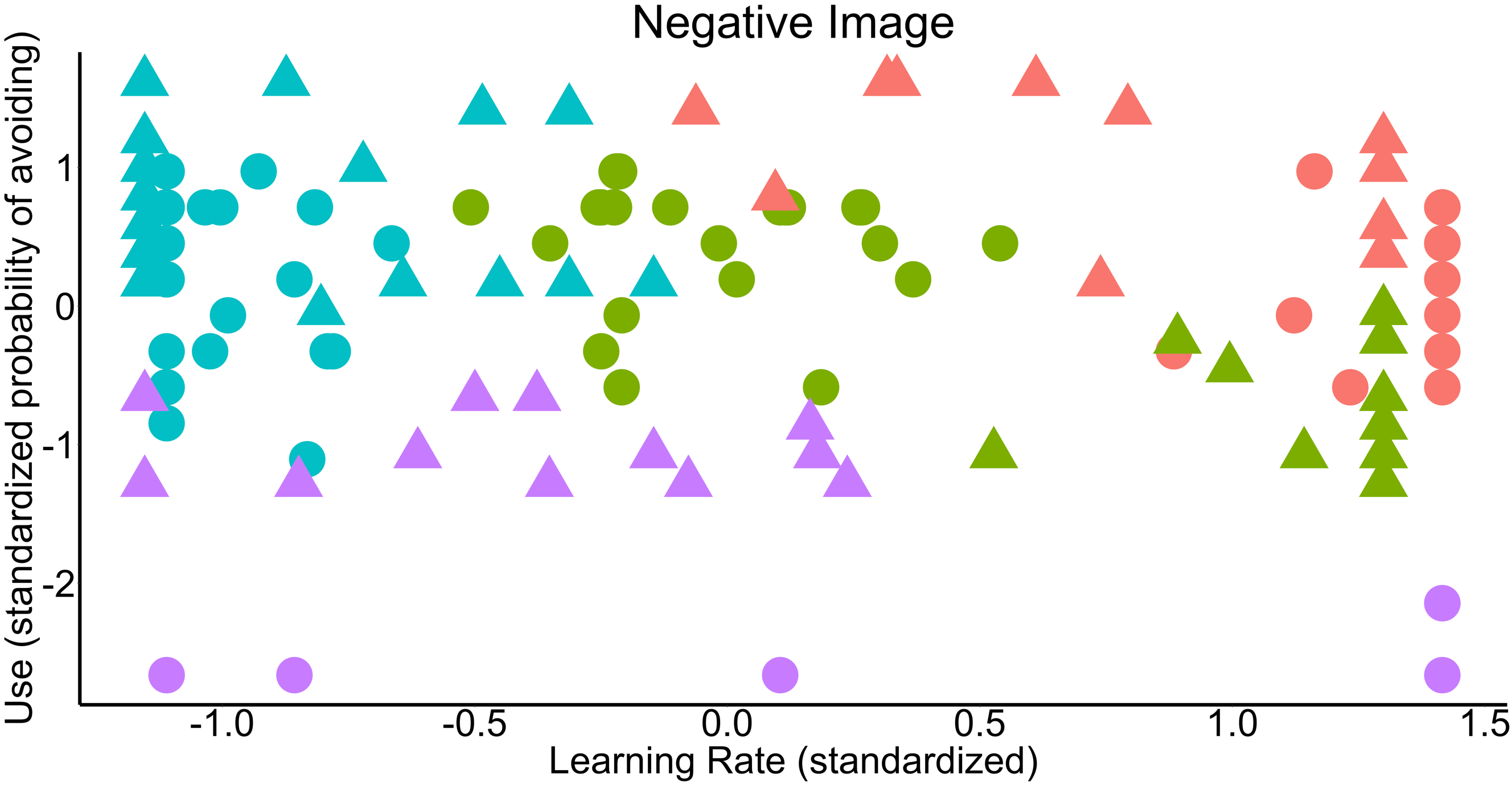


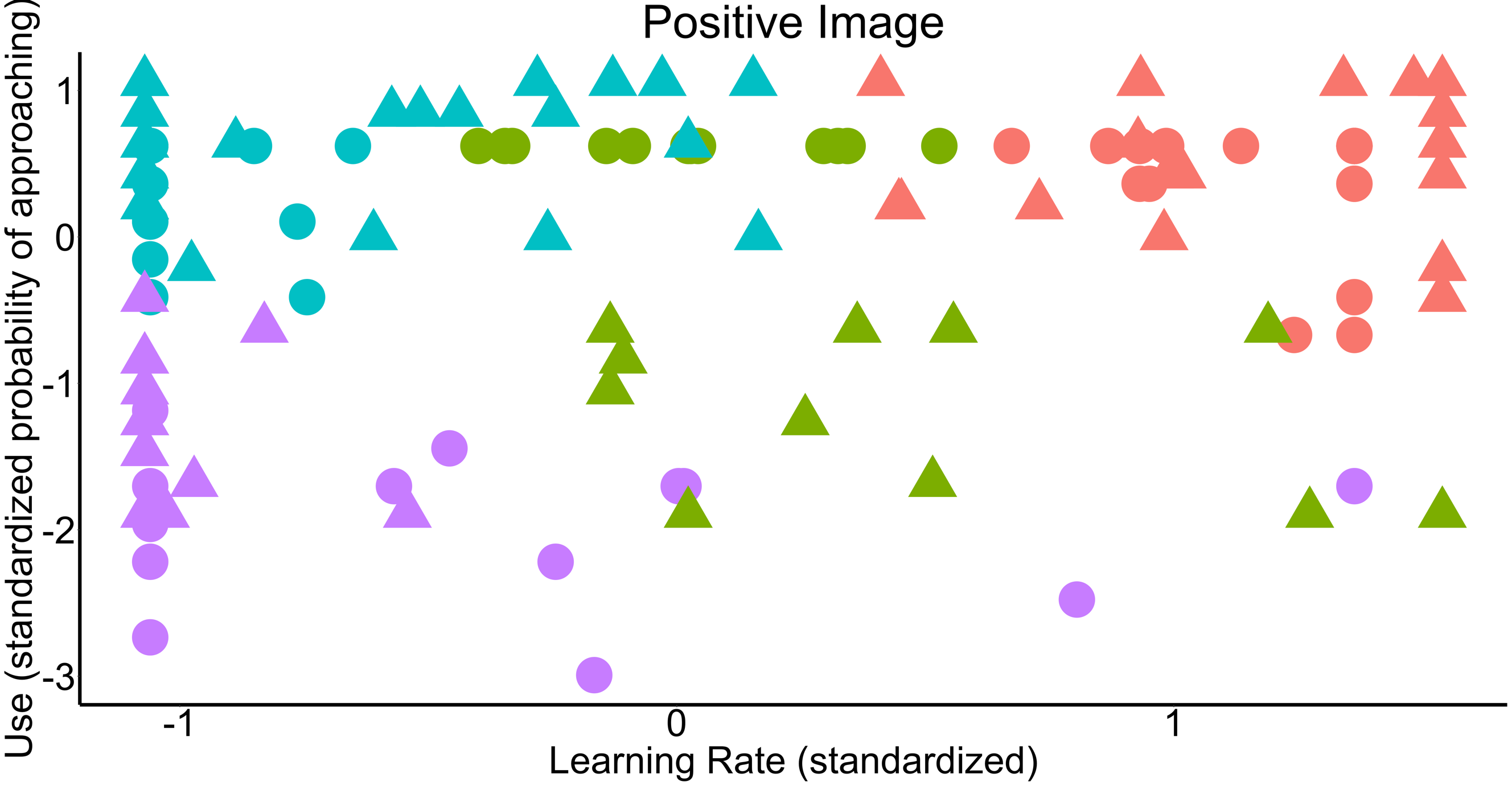


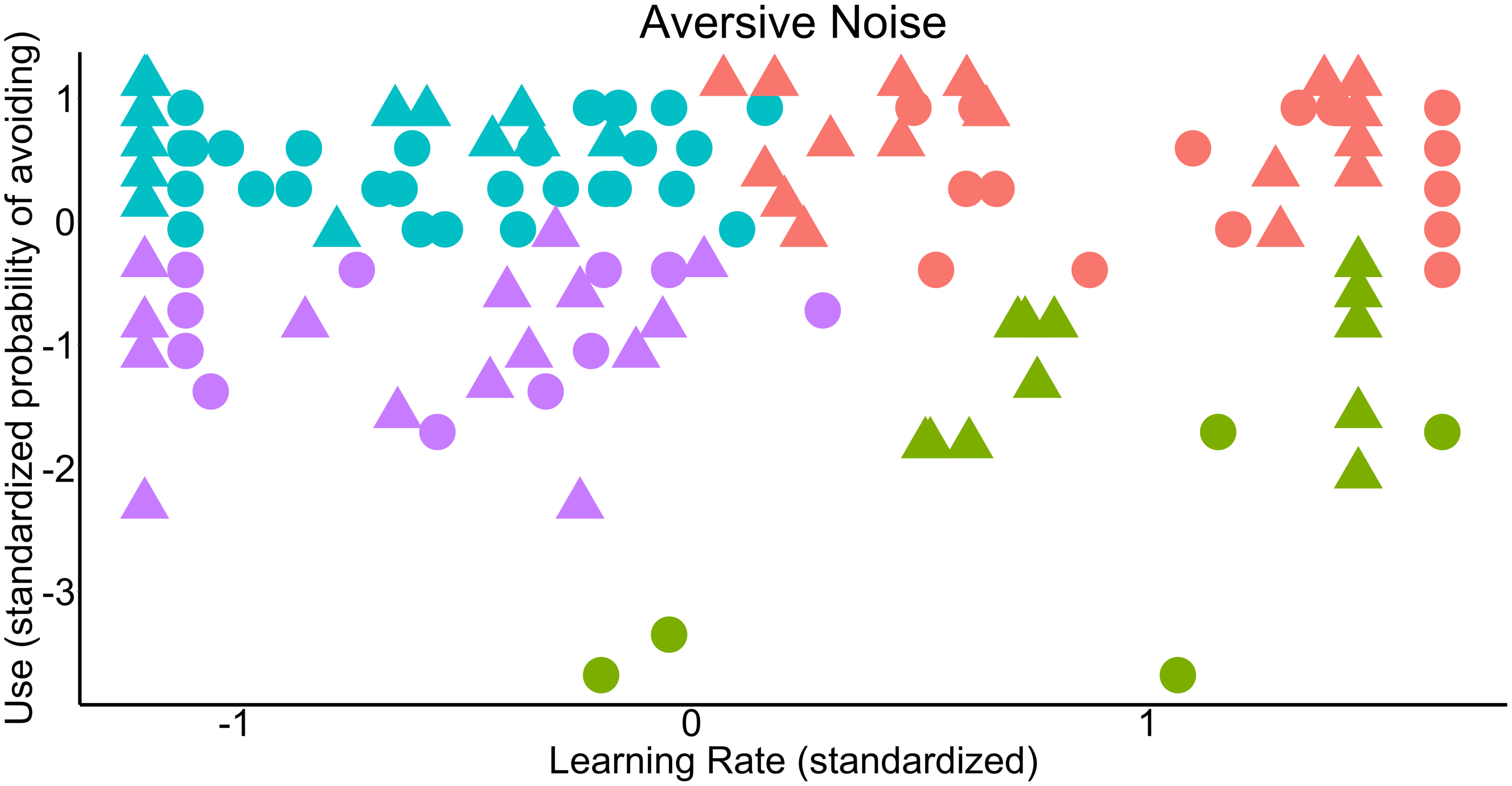


Adults

Children

**Figure S6**

*Clusters based on maximum expected value modeled using reaction times and use averaged across full trials for each reinforcer condition.*

High Value, High Use

High Value, Low Use


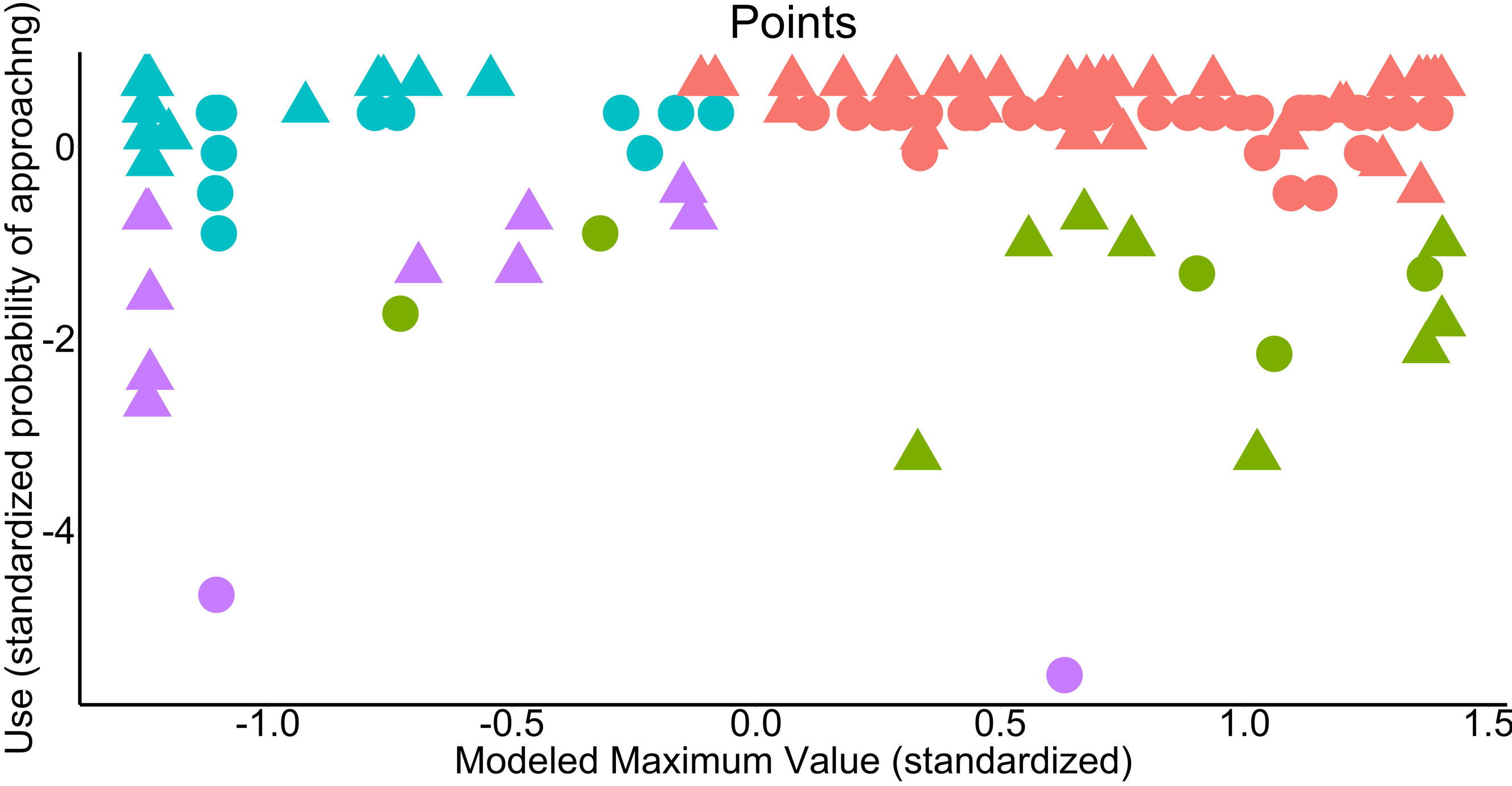


Appetite Reinforcers

Aversive Reinforcers

Low Value, Low Use

Low Value, High Use


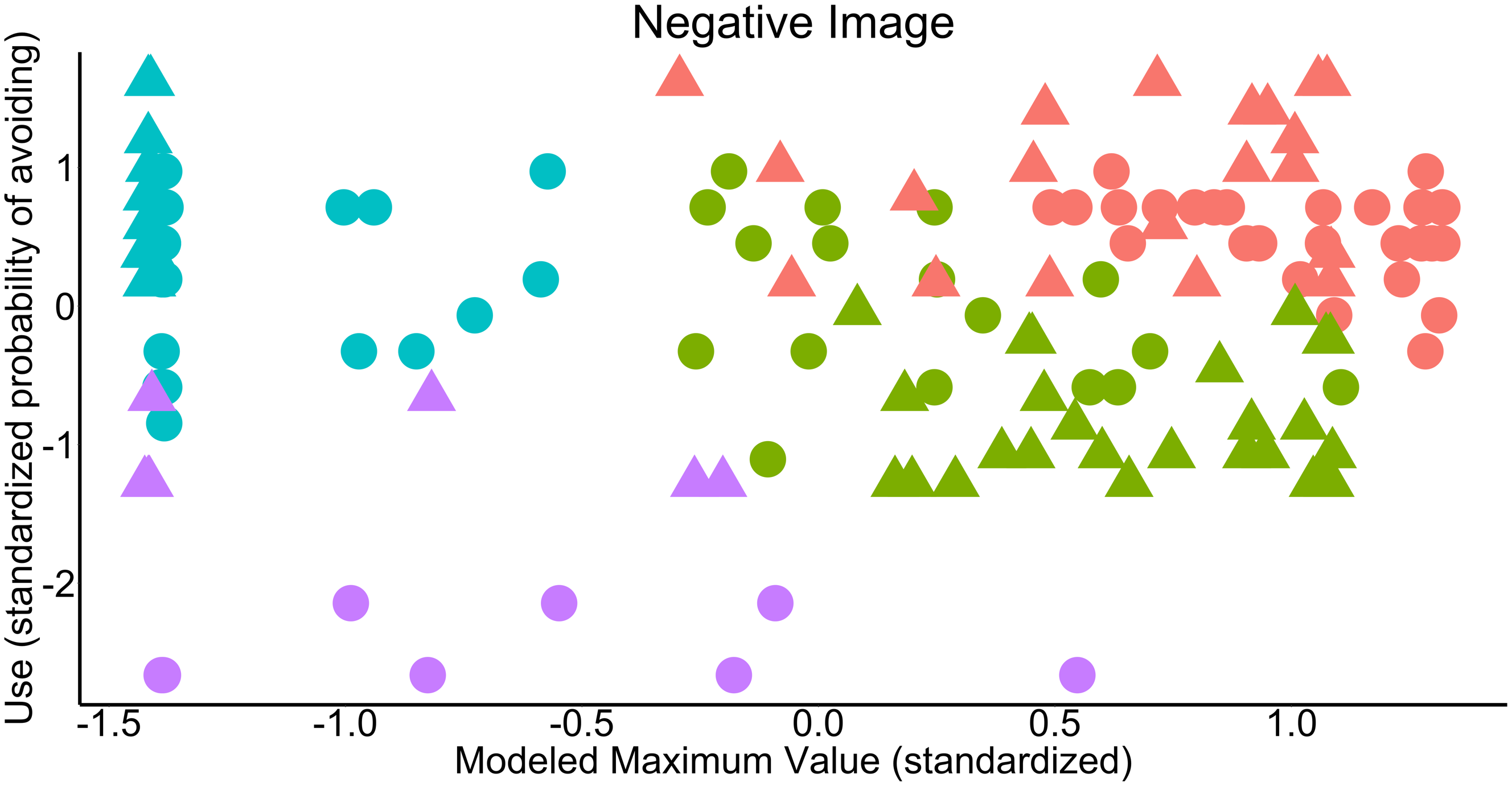


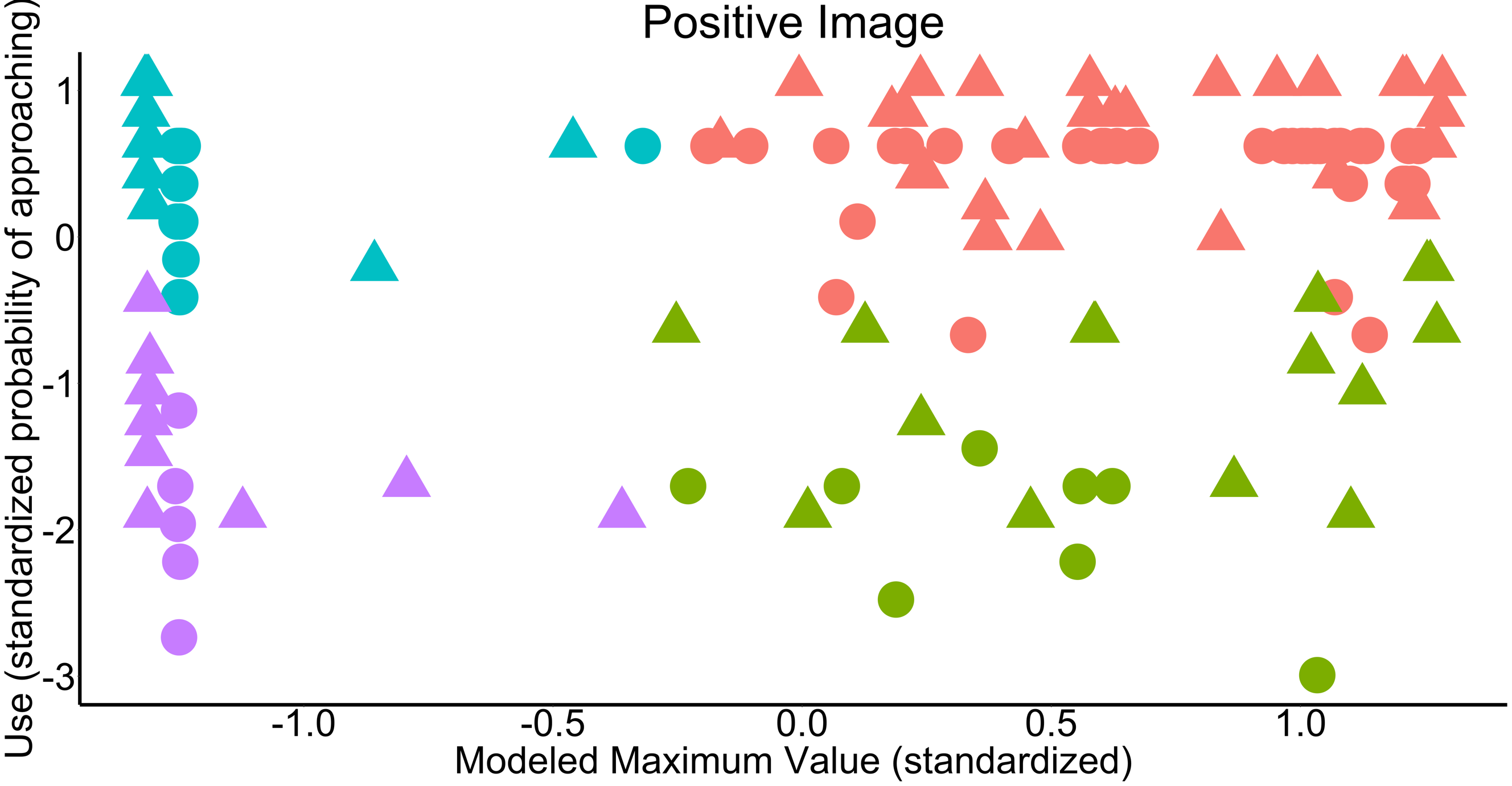


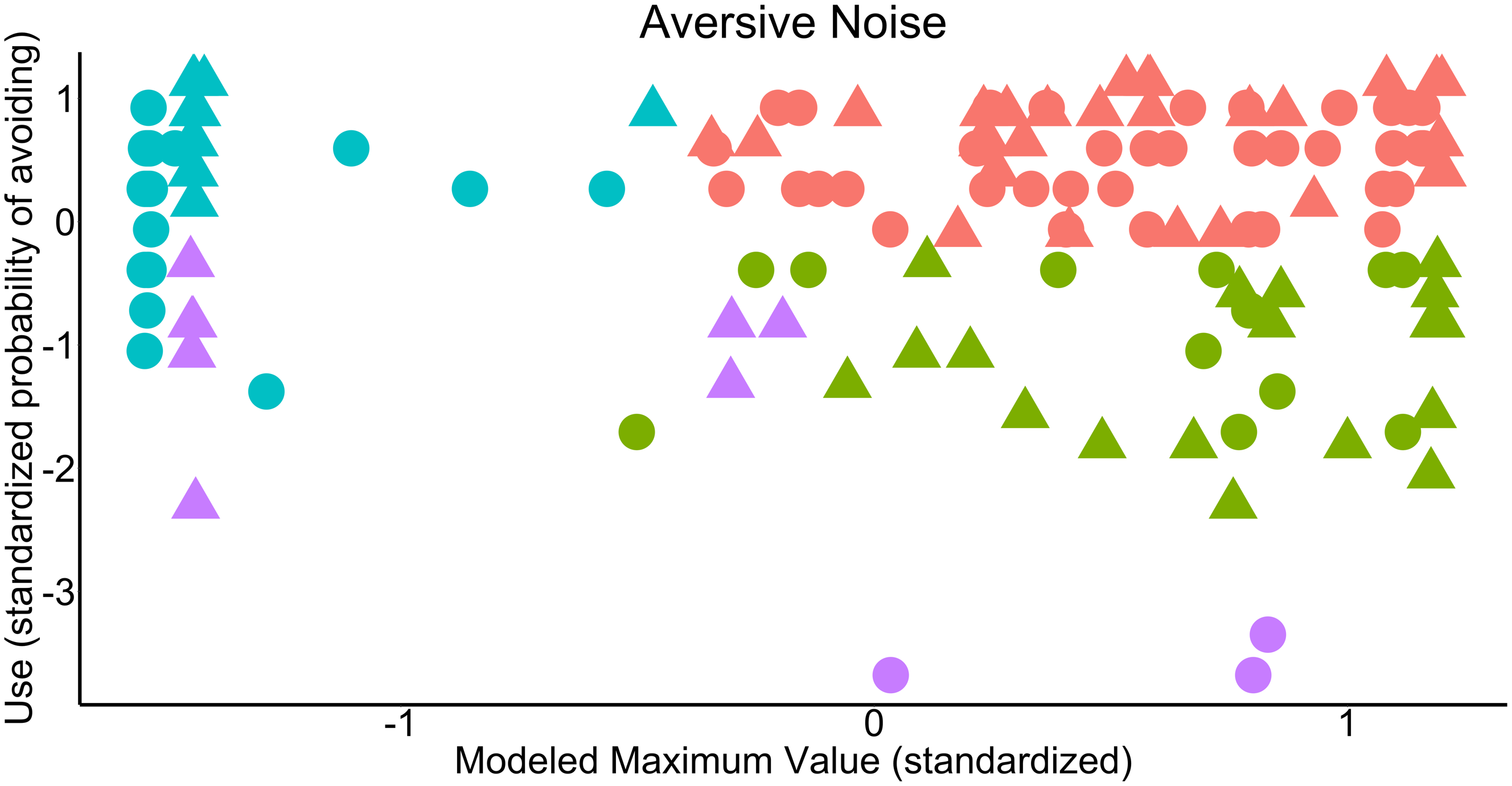


References

1. Rescorla, R. A. & Wagner, A. R. A Theory of Pavlovian Conditioning: Variations in the Effectiveness of Reinforcement and Nonreinforcement BT - Clasical conditioning II: current research and theory. *Clasical Cond. II Curr. Res. theory* 64–99 (1972).

2. Glimcher, P. W. Understanding dopamine and reinforcement learning : The dopamine reward prediction error hypothesis. *PNAS* **108**, (2011).

3. Nussenbaum, K. & Hartley, C. A. Developmental Cognitive Neuroscience Reinforcement learning across development : What insights can we draw from a decade of research ? *Dev. Cogn. Neurosci.* **40**, 100733 (2019).

4. Critchley, H. D., Mathias, C. J. & Dolan, R. J. Fear Conditioning in Humans: The influence of awareness and autonomic arousal on functional neuroanatomy. *Neuron* **33**, 653–663 (2002).

5. Gottfried, J. A., O’Doherty, J. & Dolan, R. J. Encoding Predictive Reward Value in Human Amygdala and Orbitofrontal Cortex. *Science (80-. ).* **301**, 1104–1107 (2003).

6. Seymour, B. *et al.* Temporal difference models describe higher-order learning in humans. *Nature* **429**, 664–667 (2004).

7. Metereau, E. & Dreher, J. C. The medial orbitofrontal cortex encodes a general unsigned value signal during anticipation of both appetitive and aversive events. *Cortex* **63**, 42–54 (2015).

8. Jensen, J. *et al.* Separate brain regions code for salience vs. valence during reward prediction in humans. *Hum. Brain Mapp.* **28**, 294–302 (2007).

9. O’Doherty, J. P., Buchanan, T. W., Seymour, B. & Dolan, R. J. Predictive neural coding of reward preference involves dissociable responses in human ventral midbrain and ventral striatum. *Neuron* **49**, 157–166 (2006).

10. Otto, A. R. & Daw, N. D. The opportunity cost of time modulates cognitive effort. *Neuropsychologia* **123**, 92–105 (2019).

11. Hartigan, J. & Wong, M. Algorithm AS 136 : A K-Means Clustering Algorithm. *J. R. Stat. Soc. Ser. C (Applied Stat.* **28**, 100–108 (1979).

12. Kaufman, L. & Rousseeuw, P. J. Finding groups in data: An introduction to cluster analysis. in (John Wiley & Sons, Inc, 2005).

13. Xie, W. & Richards, J. E. Effects of interstimulus intervals on behavioral, heart rate, and event-related potential indices of infant engagement and sustained attention. *Psychophysiology* **53**, 1128–1142 (2016).

14. Suchotzki, K. & Gamer, M. Effect of negative motivation on the behavioral and autonomic correlates of deception. *Psychophysiology* **56**, 1–11 (2019).

15. Van Diest, I. Interoception, conditioning, and fear: The panic threesome. *Psychophysiology* **56**, 1–27 (2019).

16. Smith, K. E. & Pollak, S. D. Approach motivation and loneliness: Individual differences and parasympathetic activity. *Psychophysiology* 1–8 (2022) doi:10.1111/psyp.14036.
